# Transcriptome Analysis of Bronchoalveolar Lavage Fluid From Children With *Mycoplasma pneumoniae* Pneumonia Reveals Natural Killer and T Cell-Proliferation Responses

**DOI:** 10.3389/fimmu.2018.01403

**Published:** 2018-06-18

**Authors:** Man Gao, Kuo Wang, Mingyue Yang, Fanzheng Meng, Ruihua Lu, Huadong Zhuang, Genhong Cheng, Xiaosong Wang

**Affiliations:** ^1^Department of Pediatrics, The First Hospital of Jilin University, Changchun, China; ^2^Institute of Translational Medicine, The First Hospital of Jilin University, Changchun, China; ^3^Department of Microbiology Immunology and Molecular Genetics, University of California Los Angeles, Los Angeles, CA, United States

**Keywords:** *Mycoplasma pneumoniae* pneumonia, bronchoalveolar lavage fluid, children, natural killer cells, CD8+ T cells, interferon gamma

## Abstract

**Background:**

*Mycoplasma pneumoniae* pneumonia (MPP) is one of the most common community-acquired pneumonia; this study is to explore the immune-pathogenesis of children MPP.

**Methods:**

Next-generation transcriptome sequencing was performed on the bronchoalveolar lavage fluid cells from six children with MPP and three children with foreign body aspiration as control. Some of the results had been validated by quantitative real-time PCR in an expanded group of children.

**Results:**

Results revealed 810 differentially expressed genes in MPP group comparing to control group, of which 412 genes including *RLTPR, CARD11* and *RASAL3* were upregulated. These upregulated genes were mainly enriched in mononuclear cell proliferation and signaling biological processes. Kyoto encyclopedia of genes and genomes pathway analysis revealed that hematopoietic cell linage pathway, natural killer cell-mediated cytotoxicity pathway, and T cell receptor signaling pathway were significantly upregulated in MPP children. In addition, significant alternative splicing events were found in *GNLY* and *SLC11A1* genes, which may cause the differential expressions of these genes.

**Conclusion:**

Our results suggest that NK and CD8+ T cells are over activated and proliferated in MPP children; the upregulated *IFNγ, PRF1, GZMB, FASL*, and *GNLY* may play important roles in the pathogenesis of children MPP.

## Introduction

*Mycoplasma pneumoniae* pneumonia (MPP) counts for 20–40% of community-acquired pneumonia in children and even higher during epidemics (1, 2). After 7 days of macrolides therapy, some children show clinical and radiological deterioration with various complications such as bronchiolitis obliterans, pulmonary necrosis, or encephalitis, some children even develop life-threatening pneumonia (3, 4). Additional treatment of corticosteroids is effective to improve the clinical symptoms in the severe cases, which suggests that the lung injuries are associated with hyperactive immune responses of host against *Mycoplasma pneumoniae* (MP) infection (5–8). However, the immune-pathogenesis of MPP remains to be elucidated. Studies based on the serum cytokines demonstrate that CD4+ T cells are involved in the development of MPP (9, 10), but little evidence has been found to support that other T cell subsets or natural killer (NK) cells are involved in the pathogenesis of MPP.

Next-generation sequencing is independent on the predetermined genome sequences, highly accurate with wide dynamic detection ranges and low background (11, 12). Therefore, using this method, we analyzed the transcriptome of bronchoalveolar lavage fluid (BALF) from children with MPP and children with airway foreign body aspiration (FB) as control (Additional File 1: Table S1 in Supplementary Material). Comparing to peripheral blood, BALF can better reflect the local bronchoalveolar immune responses. We found that local proliferation responses of NK cells and CD8+ T cells had increased in MPP children comparing to control, which revealed that both NK cells and CD8+ T cells played important roles in the pathogenesis of children MPP.

## Materials and Methods

### Study Subjects

This study was conducted at the First Hospital of Jilin University (Changchun City, Jilin Province, People’s Republic of China). Children with acute MPP were recruited; children with FB were recruited as control. The diagnosis of FB relied on airway foreign body aspiration history and bronchoscopy findings. The diagnosis of pneumonia was based on clinical manifestations (cough, fever, dry, or productive sputum, dyspnea, abnormal breath sound, radiological pulmonary abnormalities, etc.). The diagnosis of MP infection was based on positive results of serologic test (MP-IgM positive and antibody titer ≥ 1:40) and positive quantitative real-time PCR (qRT-PCR) results of MP deoxyribonucleic acid (DNA) (>500 copies/l) in BALF. MPP children with other respiratory tract infections were excluded by following tests: protein purified derivative, blood cultures, plural effusion cultures, nasopharyngeal aspirate/swab cultures, serology for *Chlamydia* pneumonia (CT), serology for *Legionella pneumophila* (LG), and serology detection for virus antigens (respiratory syncytial viruses, influenza viruses, metapneumovirus, adenovirus, and parainfluenza virus). Children who received corticosteroids before admission or had underlying diseases such as asthma, recurrent respiratory tract infection chronic cardiac and pulmonary diseases, or immunodeficiency were also excluded.

### Bronchoscopy and Bronchoalveolar Lavage (BAL)

The guidelines of bronchoscopy and BAL were described previously (13, 14). For MPP children, bronchoscopy was performed within 3 days after hospital admission, BALF was harvested within 1 week after onset of the pneumonia; for FB children, bronchoscopy was performed immediately after hospital admission to remove the brochus FB, BALF samples for sequencing was collected at the time of re-examination of bronchus FB, which is usually 1 week after the remove of bronchus foreign body. Sterile saline (0.3–0.5 ml/kg) was instilled through the instrumentation channel; BALF was gently aspirated, and collected in a sterile container, filtered and centrifuged. The pallet was resuspended in TRIzol (Life Technologies, CA, USA) and stored in −80°C freezer. Lymphocyte profiles in the BALF samples from MPP children and FB control children were examined (Additional File 2: Table S2 in Supplementary Material). Results showed that absolute cell numbers per microliter of NK cells, B cells, CD4+ T cells, and CD8+ T cells were significantly higher in BALF of MPP children comparing to that of FB children. However, no significant difference was found in the cellular composition of NK cells, B cells, CD4+ T cells, and CD8+ T cells in total lymphocytes between the two groups.

### Isolation of Ribonucleic Acid (RNA) and RNA Sequencing

Total RNA was extracted using TRIzol according to the manufacturer’s instructions. RNA integrity was assessed using the RNA Nano 6000 Assay Kit of the Bioanalyzer 2100 system (Agilent Technologies, CA, USA). 3 µg RNA per sample was used as input material for further analysis. NEBNext^®^ Ultra™ RNA Library Prep Kit was used to generate sequencing libraries for Illumina^®^ (NEB, USA) based on the manufacturer’s recommendations; index codes were added to attribute sequences to each sample. Through a cBotCluster Generation System, the clustering of the index-coded samples was performed with TruSeq PE Cluster Kit v3-cBot-HS (Illumia) following the manufacturer’s instructions. After cluster generation, the library preparations were sequenced on an Illumina Hiseq platform, and 125/150 bp paired-end reads were generated.

### Sequencing Data Analysis

Raw data (raw reads) of fastq format were first processed through in-house perl scripts. In this step, clean data (clean reads) were obtained by removing reads containing adapter, reads containing poly-N and low-quality reads from raw data. All the downstream analyses were based on the clean data with high quality. Index of the reference genome was built using Bowtie v2.2.3, and paired-end clean reads were aligned to the reference genome using TopHat v2.0.12. The differentially expressed genes between MPP group and control group were identified using the DESeq R package (1.18.0) (15). HTSeq v0.6.1 was used to count the reads numbers mapped to each gene. And then fragments per kilobase of transcript sequence per million base pairs sequenced (FPKM) of each gene was calculated based on the length of the gene and reads count mapped to this gene (16). The *p* values were adjusted using Benjamini and Hochberg’s approach for controlling the false discovery rate (FDR). Gene with an adjusted *p* value < 0.05 was assigned as differentially expressed. Unsupervised heat maps of clustering analysis were generated by normalizing all data between maximum (red) and minimum (blue) for each individual reads using Cluster 3.0.

Gene ontology (GO) enrichment analysis of the differentially expressed genes was implemented by the GOseq R package, in which gene length bias was corrected. GO terms with adjusted *p* value < 0.05 were considered significantly enriched. We used KOBAS software to test the statistical gene enrichment of the differentially expressed genes in Kyoto encyclopedia of genes and genomes (KEGG) pathways. Protein–protein interaction (PPI) analysis of differentially expressed genes was based on the Search Tool for the Retrieval of Interacting Genes/Proteins (STRING) database v10.0, the minimum STRING score was set at 700 (highest confidence). Cytoscape software was applied to visualize the protein network. Connectivity threshold values for hub genes were means + 2 SDs.

The Cufflinks v2.1.1 Reference annotation-based transcript assembly method was used to identify both known and novel transcripts from TopHat alignment results. Alternative splicing events were classified to five types by software Asprofile v1.0. The analysis of alternative splicing events was performed using MATS and integrative genomics viewer software (17); the differences in alternative splicing of genes were considered significant with a cutoff of 5% FDR.

### Validation of the Upregulated Genes in the KEGG Pathways

Quantitative real-time PCR analysis was performed to validate the upregulated genes in three KEGG pathways. qRT-PCR was carried out as described previously (14, 18). Briefly, total RNA was extracted using TRIzol reagent; complementary DNA was synthesized using the Prime Script RT Reagent Kit (TAKARA, Kyoto, Japan) and amplified using the Fast start universal SYBR green master (Roche Diagnostics GmbH, Mannheim, Germany). Each sample was assayed in duplicate; every Ct value was the average of results from two wells. Glyceraldehyde-3-phosphate dehydrogenase (GAPDH) was selected as the reference gene. The method of 2^−ΔΔCt^ was used to analyze qRT-PCR data, which was expressed as the fold-change relative to the value of GAPDH (19, 20). Statistical analyses of qRT-PCR results were performed using GraphPad 5.0. The classification data was analyzed by chi-squared tests. The comparisons were carried out with the Mann–Whitney *U* test. Statistical significance was defined as *p* < 0.05.

## Results

### Clinical Characteristics of the Sequencing Subjects

**Figure 1:**
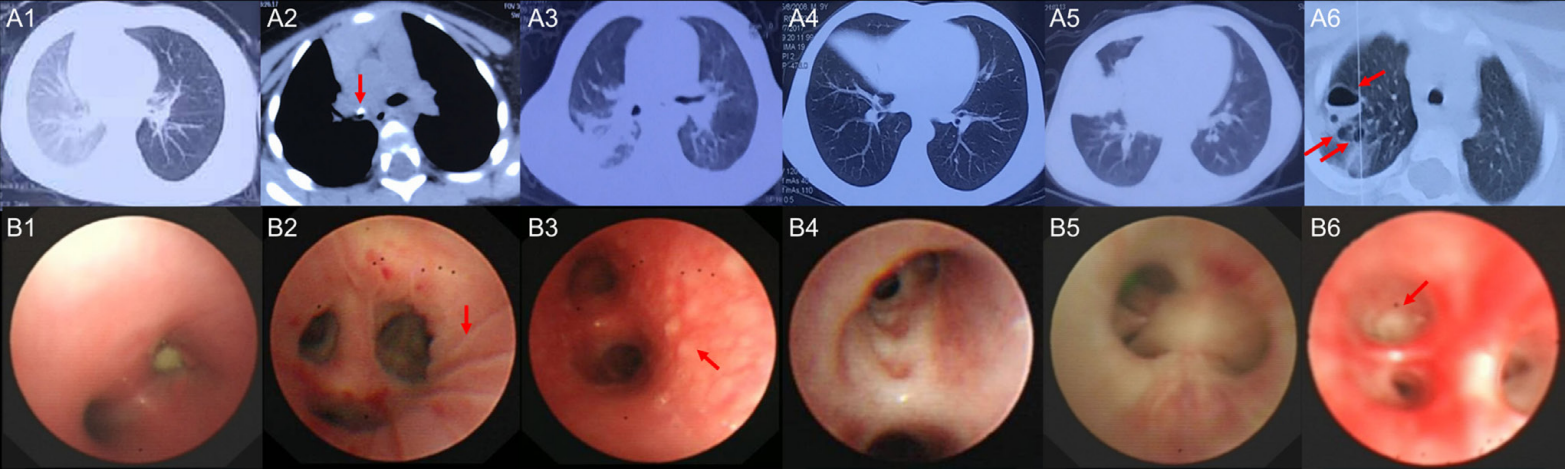
The radiological images and bronchoscopic images of the subjects enrolled in sequencing. **(A1)** shows radiological image of control1, emphysema (air trapping) in the left lung was observed; **(A2)** shows radiological image of control 3, foreign body (red arrow) blocked the right main bronchus; **(A3)** shows radiological image of *Mycoplasma pneumoniae* pneumonia (MPP) 1, patchy density shadows in the right lung were observed; **(A4)** shows radiological image of MPP3, atelectasis of the right middle lobe was found; **(A5)** shows radiological image of MPP4, which revealed large density shadows (consolidation) in the right middle lobe; **(A6)** shows radiological image of MPP6, multiple necrotizing sacs (red arrows) were observed in the right lung. The wall of the sacs was very thin, which was different from the lung abscess; **(B1)** is the bronchoscopic image of control 2, a peanut was observed in the right main bronchus, which blocked the airway; **(B2)** is the bronchoscopic image of MPP2, lines on mucosa (red arrow) were observed in the right upper lobe; **(B3)** is the bronchoscopic image of MPP5, which shows diffused mucosal nodules (red arrow) in distal part of the left main bronchus; **(B4)** is the bronchoscopic image of MPP6, which shows the erosion of mucosa and secretions in the bronchus; **(B5)** is the bronchoscopic image of MPP4, which shows sputum plugging; **(B6)** is the bronchoscopic image of MPP3, the red arrow shows the proliferation of fibrous tissue occluded the bronchus orifice completely.

Six children with acute MPP and three children with FB (Additional File 1: Table S1 in Supplementary Material) were recruited for sequencing. FB children had no fever, the white blood cell (WBC) counts and C-reaction protein (CRP) levels were within normal range, the radiological images showed air trapping or opaque object (Figures 1A1, A2), the bronchoscopic image did not show obvious inflammation changes (Figure 1B1) for FB children. MPP children had fever, normal WBC counts, and increased CRP. MPP children had radiologically proven large pulmonary lesions including patchy density shadows, atelectasis, consolidation, or necrotizing sacs (Figures 1A3–A6), their bronchoscopic images showed mucosal lines, mucosal nodules, mucosal erosion and secretions, sputum plugging, and proliferation of fibrous tissue (hyperplasia membrane) (Figures 1B2–B6). Comparing to FB children, these images of MPP children suggested acute lung injuries. After BALF collection, the composition of the nucleated cells was counted (Additional File 3: Table S3 in Supplementary Material). Results showed that nucleated cells in BALF were mainly macrophages, neutrophils and lymphocytes. The absolute cell numbers per liter are higher in the BALF of MPP children comparing to that of FB children. However, there is NO significant difference found in the cellular composition between the two groups.

### RNA Sequencing Results

**Figure 2:**
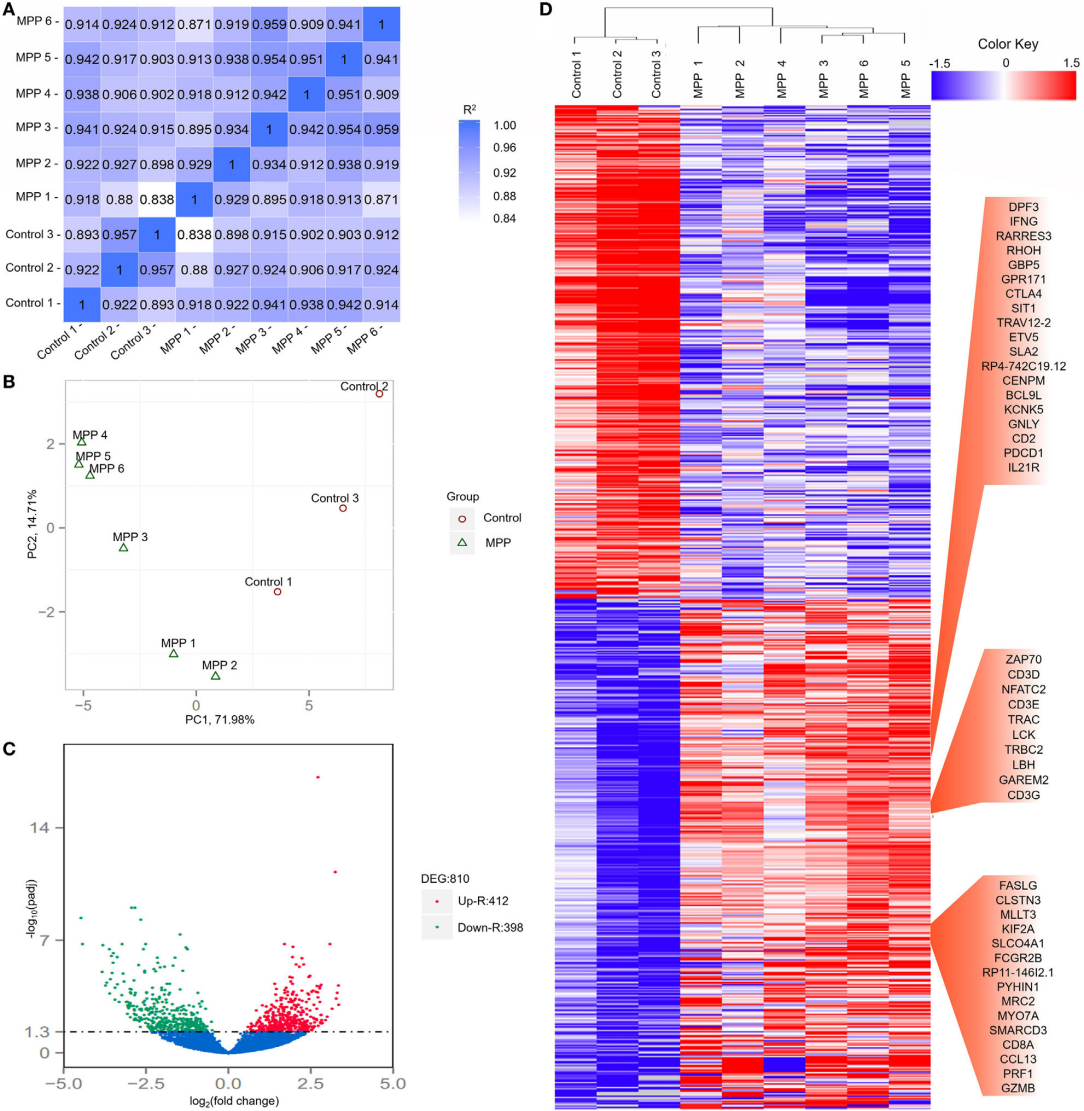
Evaluation of each bronchoalveolar lavage fluid sample included in this study and differentially expressed genes between *Mycoplasma pneumoniae* pneumonia (MPP) group and control group. **(A)** The correlation coefficient heat map of MPP group and control group. Correlation matrix shows a high consistency of measurements within each group, *R*^2^ ≥ 0.8 is needed for the up-coming analyzing. **(B)** Principal component analysis (PCA) plot of the sequencing samples. PCA is conducted to evaluate the clustering nature of the samples. The repeatability of the samples has been shown. Each point represents one sample, the red circles represent the samples in the MPP group, and the green triangles represent the samples in the control group. Percentages are contribution ratios. **(C)** Volcano plot of genes differentially expressed between MPP group and control group. Each point represents one gene that is detectable in both groups. The red points represent the significantly upregulated (Up-R) genes; the green points represent the significantly downregulated (Down-R) genes. **(D)** Cluster of 810 genes showing significantly regulated genes between MPP group and control group. All of the genes that are differentially expressed between MPP group and control group by adjusted *p* value < 0.05 have been selected.

To investigate the gene changes related to the acute lung injuries of MPP children, total RNA was extracted from BALF samples of each child. Following sequencing, adaptor sequences, ambiguous reads, and low-quality reads were removed, and about 40–70 million pairs of clean reads were generated for each sample (Additional File 4: Table S4 in Supplementary Material). Comparing with the reference sequence of the Genome reference consortium GRCh37/hg19, more than 84% of total read pairs were uniquely mapped on the human genome. Mapped reads were used to estimate normalized transcription levels as FPKM. A correlation matrix showed a high consistency of measurements within each group, *R*^2^ > 0.8 (Figure 2A). Principal component analysis (PCA) was carried out to assess the clustering nature of these samples. The samples of each group had been clustered together, data shown good repeatability and correlation (Figure 2B).

### Identification and Classification of Differentially Expressed Genes Between MPP Group and Control Group

Totally 810 differentially expressed genes (412 upregulated genes and 398 downregulated genes) were identified between the MPP group and control group (Figure 2C; Additional File 5: Table S5 in Supplementary Material). Clustering analysis results are shown in Figure 2D. Some upregulated genes that had similar expression patterns were listed in red panel groups, which were interferon gamma (IFNγ)/RHOH/granulysin (GNLY)/CD2/IL21R panel, ZAP70/CD3D/NFATC2/CD3E/LCK/CD3G panel, and FASLG/CD8A/PRF1/granzyme B panel. The top 20 upregulated genes (Additional File 5: Table S5 in Supplementary Material) were TBX21, CD40LG, ZBED2, PDCD1, IL21R, CD2, IL32, AC092580.4, CD3D, NCALD, IL12RB2, TNFRSF4, TIFAB, IL2RB, CD247, GFI1, FDRL6, FGFBP2, IFNγ, and KIR2DL3.

**Figure 3:**
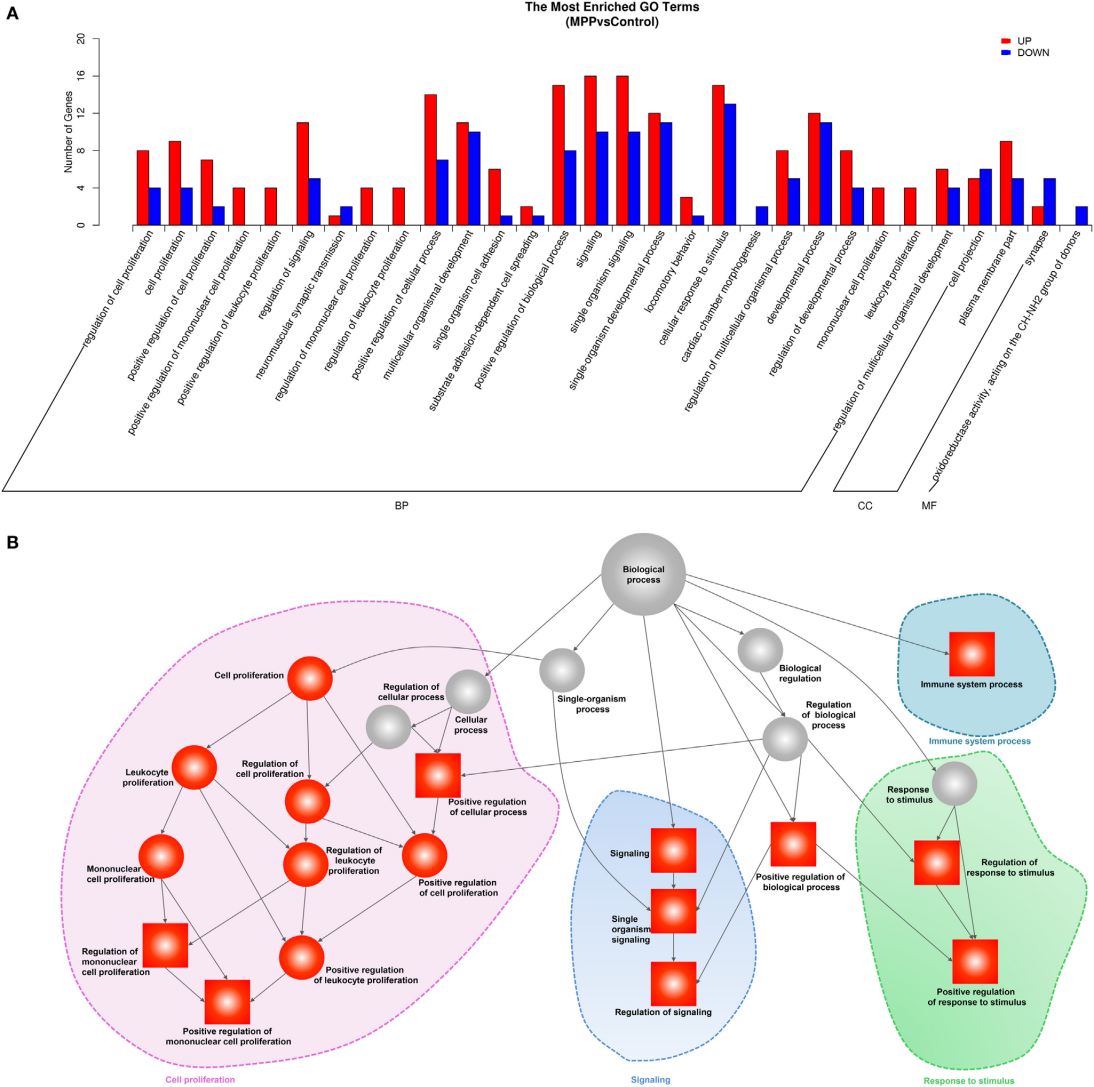
Gene ontology (GO) analysis of the deferentially expressed genes between *Mycoplasma pneumoniae* pneumonia (MPP) group and control group. **(A)** Top 30 GO terms were enriched as biochemical processes (BP), cellular components (CCs), or molecular function (MF). The numbers of deferentially expressed genes between MPP and control in each category were compared. Red bars represent upregulated genes, and blue bars represent downregulated genes. **(B)** Directed acyclic graph of GO enrichment of upregulated genes, the square represents the top 10 GO terms based on adjusted *p* values; red squares or red circles represent higher degrees of enrichment.

To explore the biological functions of these differentially expressed genes, we performed GO analysis based on the GO annotation terms. As shown in Figure 3A, enriched GO terms were classified to biological process (BP) class, cellular component class and molecular function (MF) class, 26 out of the top 30 GO terms differently enriched between MPP and control belonged to the cell proliferation and signaling terms of BP class (Additional File 6: Table S6 in Supplementary Material). Furthermore, the upregulated genes of BP class were depicted as directed acyclic graphs in Figure 3B, which showed the relationships of the GO terms. The adjusted *p* value, degree of enrichment, and involved gene names of each significantly increased GO term were listed in Additional File 7: Table S7 in Supplementary Material. The significantly upregulated genes including caspase recruitment domain family member 11 (CARD11), RLTPR, and RAS protein activator like 3 (RASAL3) were highly enriched into 4 GO terms, which were immune system process (GO:0002376), regulation of signaling (GO:0023051), positive regulation of mononuclear cell proliferation (GO:0032946), and positive regulation of response to stimulus (GO:0048548).

### KEGG Pathway Enrichment Analysis

**Figure 4:**
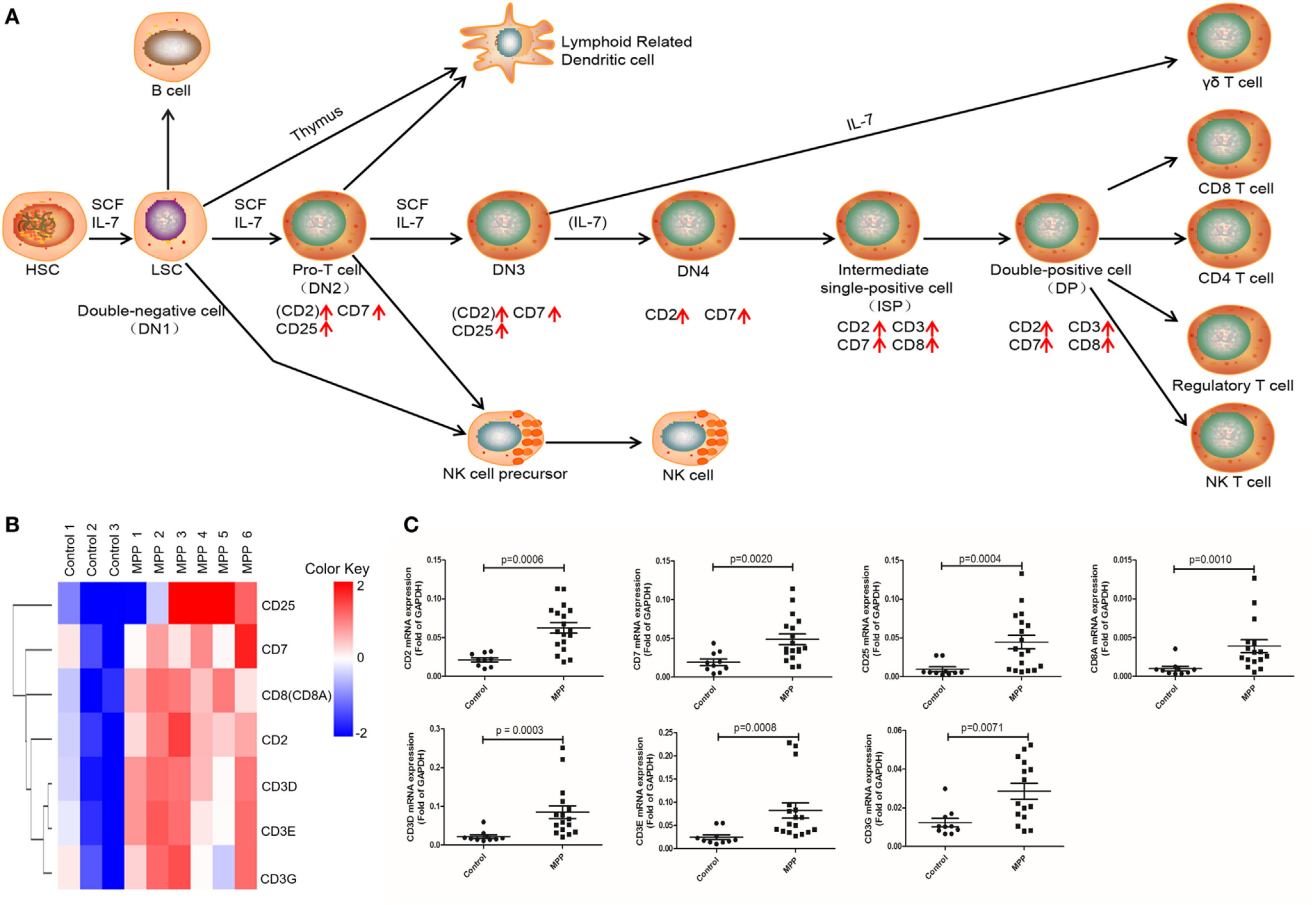
Differential expression genes (DEGs) mapped to hematopoietic cell lineage Kyoto encyclopedia of genes and genomes pathway. **(A)** Model diagram shows hematopoietic cell lineage pathway, red arrows indicate upregulated gene expression. **(B)** Cluster analysis of DEGs that mapped to hematopoietic cell lineage pathway. **(C)** Quantitative real-time PCR validation of DEGs mapped to hematopoietic cell lineage pathway.

Gene ontology analysis revealed that the upregulated genes were highly enriched to proliferation and signaling related GO terms in MPP children. Correspondingly, KEGG analysis significantly identified three pathways (Additional File 8: Table S8 in Supplementary Material) that were highly related with mononuclear cell proliferation and signaling. These pathways were T cell receptor signaling, NK cell-mediated cytotoxicity and hematopoietic cell linage. The upregulated genes (CD25, CD7, CD8A, CD2, CD3D, CD3E, and CD3G) were involved in hematopoietic cell linage pathway (Figure 4A). These genes were involved in the differentiation process from pro-T cell to double positive T cell and finally to CD8+ T cell. These results suggested that the proliferation of CD8+ T cells were upregulated in MPP children comparing to control children. Figure 4B is the clustering analysis results of these genes, the expression patterns of CD8A and CD2 were close to that of CD3D, CD3E, and CD3G, which were consistent with the KEGG results. CD8A is an important marker of cytotoxic lymphocytes, protein network (Additional File 9: Figure S1A in Supplementary Material) showed that IL2RA, CD3D, CD3E, CD3G, CD2, and CD7 genes were the first neighbors of CD8A. Furthermore, the upregulation of CD2, CD7, and CD25 might increase the differentiation of NK cell precursor (Figure 4A). Based on these KEGG results, we could deduce that the differentiation of NK cells and CD8+ T cells were upregulated in MPP children.

**Figure 5:**
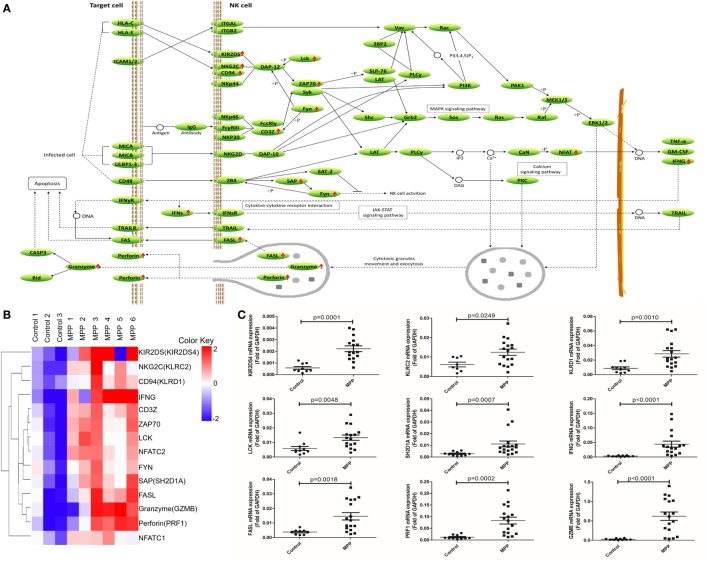
Differential expression genes (DEGs) mapped to natural killer (NK) cell-mediated cytotoxicity Kyoto encyclopedia of genes and genomes pathway. **(A)** Model diagram showing NK cell-mediated cytotoxicity pathway, red arrows indicate upregulated gene expression. **(B)** Cluster analysis of DEGs that mapped to NK cell-mediated cytotoxicity pathway. **(C)** Quantitative real-time PCR validation of DEGs mapped to NK cell-mediated cytotoxicity pathway.

To further explore this hypothesis, first, the upregulated genes [KIR2DS, NKG2C, CD94, IFNγ, CD3Z, ZAP70, LCK, NFAT, FYN, SAP, Fas ligand (FASL), granzyme, and perforin] were mapped to the nature killer cell-mediated cytotoxicity KEGG pathway (Figure 5A). Infected cells stimulated NK cells and upregulated their surface molecules including KIR2DS, NKG2C, CD94, and CD3Z, followed by the upregulation of LCK, ZAP70, and FYN. Upregulated NFAT entered the nucleus of NK cells to upregulate their IFNγ expression. Cytotoxic granules in the cytoplasm of NK cells moved to release FASL, granzyme, and perforin. IFNγ combined with IFNγR and further activated FAS on the infected cell, the combination of FASL and FAS delivered cell-death signal to infected cells. In addition, upregulated perforin induced the perforation of host cells, and then granzyme entered and induced the apoptosis of infected cells. Clustering analysis (Figure 5B) suggested that the expression pattern of granzyme was most close to perforin and FASL. Protein network analysis confirmed that perforin and FASL were the first neighbors of granzyme (Additional File 9: Figure S1B in Supplementary Material).

**Figure 6:**
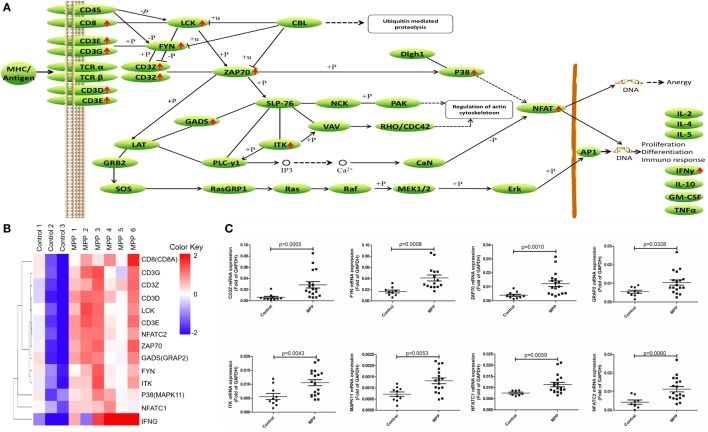
Differential expression genes (DEGs) mapped to T cell receptor signaling pathway Kyoto encyclopedia of genes and genomes pathway. **(A)** Model diagram showing T cell receptor signaling pathway, red arrows indicate upregulated gene expression. **(B)** Cluster analysis of DEGs that mapped to T cell receptor signaling pathway. **(C)** Quantitative real-time PCR validation of DEGs mapped to T cell receptor signaling pathway.

Second, the upregulated genes (CD8, CD3G, CD3Z, CD3D, LCK, CD3E, NFAT, ZAP70, GADS, FYN, ITK, P38, and IFNγ) were mapped to T cell receptor signaling KEGG pathway (Figure 6A). After infection, MP antigens stimulate and upregulate T cell surface molecules including CD3D, CD3E, CD3G, and CD8, followed by the upregulation of downstream molecules including CD3Z, FYN, LCK, ZAP70, GADS, P38, and ITK. Once NFAT were activated, it entered the nucleus of T cells to activate the transcription of IFNγ. Clustering analysis (Figure 6B) suggested that IFNγ was closely related with other genes in this pathway. Protein network analysis confirmed that NFATC1, NFATC2, ZAP70, CD8A, CD3D, CD3E, CD3G, and CD3Z were the first neighbors of IFNγ (Additional File 9: Figure S1C in Supplementary Material).

To validate these results, 24 upregulated genes involved in these three KEGG pathways were detected by qRT-PCR. BALF samples were collected from FB children and MPP children (Additional File 10: Table S9 in Supplementary Material). The specific primers (Sangon, Shanghai, China) were listed in Additional File 11: Table S10 in Supplementary Material. As shown in Figures 4C, 5C and 6C, the expression levels of these genes in MPP children were significantly higher compared with control children. Therefore, 24 upregulated genes suggested by RNA sequencing had been confirmed by qRT-PCR method.

The transcriptomic data on other cell types was shown in Additional File 12: Figure S2 in Supplementary Material. Genes including SCF, IL7, CD34, CD135, Flt3L, G-CSF, IL3, IL6, IL11, GM-CSF, and HLA-DR were involved in the differentiation of mast cells, basophils, eosinophils, and myeloid-related dendritic cells. Results showed that no significant difference was found on those genes between MPP group and FB control group. CD15 is in white square, which indicated that it was not detectable. Therefore, we did not have evidence to say that the differentiation of macrophages, eosinophils, and neutrophils was upregulated in MPP group comparing to control group.

### Alternative Splicing Events Between MPP Group and Control Group

**Figure 7:**
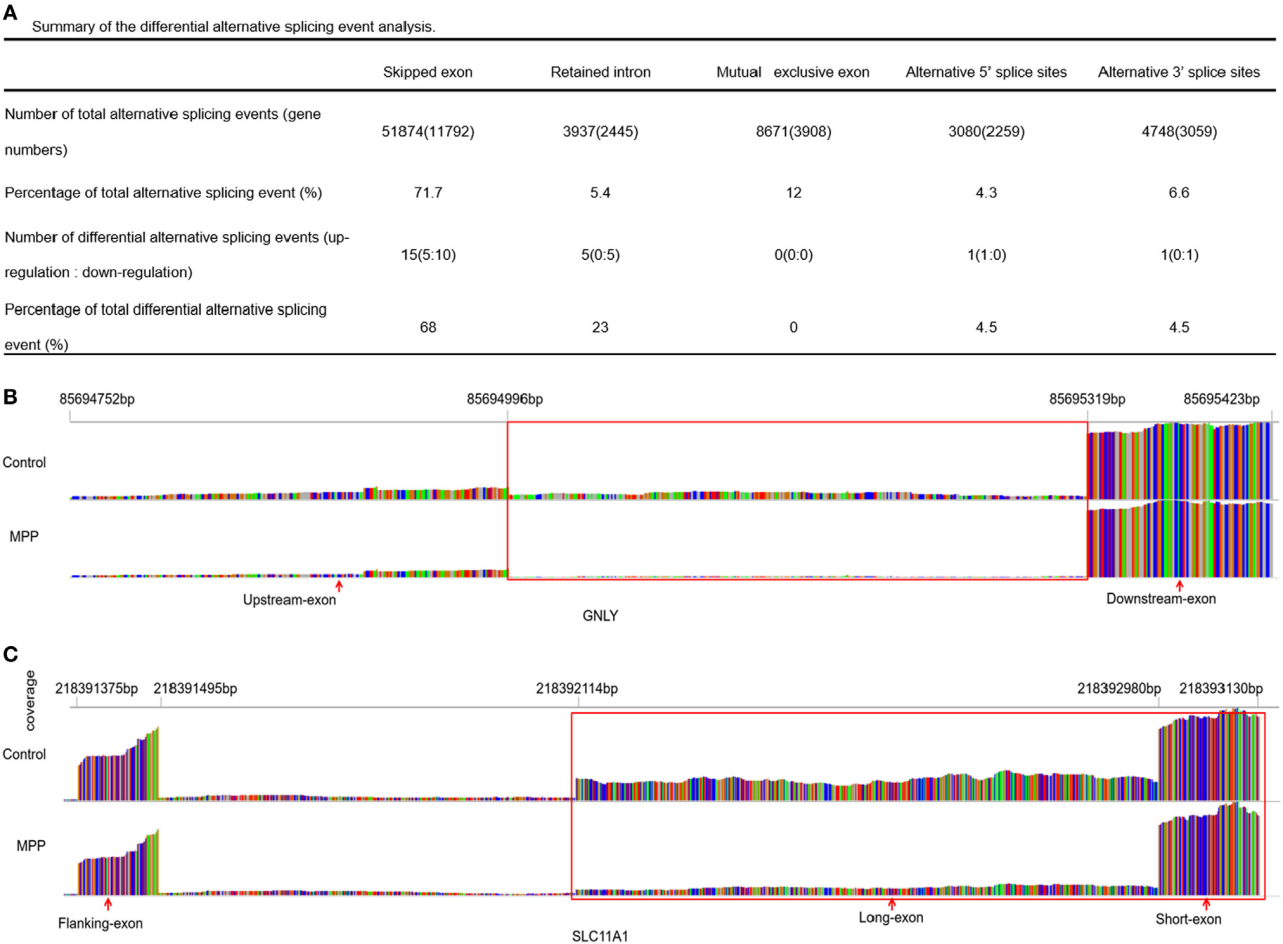
Differential alternative splicing events. **(A)** Summary of the differential alternative splicing event analysis results. **(B)** Read distribution plot for granulysin (GNLY) with differential isoform expression due to retained intron between *Mycoplasma pneumoniae* pneumonia (MPP) group and control group. **(C)** Read distribution plot for solute carrier family 11 (SLC11A1) with differential isoform expression due to alternative 3′ splice sites (A3SS) between MPP group and control group.

More than 90% of human genes are alternatively spliced through different types of splicing (21). Comparing with control, MATS analysis revealed 22 significantly differential alternative splicing events in MPP children (Figure 7A). 15 of them (68%) belonged to skipped exon. Among the differentially expressed genes between MPP and control, GNLY and solute carrier family 11 (SLC11A1) were identified to have significant alternative splicing (Additional File 13: Table S11 in Supplementary Material).

Granulysin is located on 2p11.2 (chr2: 85,685,175–85,698,854). GNLY encodes a member of saposin-like protein (SAPLIP) family, which locates in the cytotoxic granules of T cells and is released upon antigen stimulation. Retained intron (RI, chr2: 85,694,752–85,695,423) was identified in GNLY (Figure 7B). The RI levels of GNLY in MPP were lower than that of the control, which may explain the upregulation of GNLY in MPP children.

Solute carrier family 11 is located on 2q35 (chr2: 218,381,766–218,396,894). SLC11A1 encodes a divalent transition metal transporter involved in iron metabolism and host resistance to certain pathogens. Alternative 3′ splice site (A3SS) was identified in SLC11A1 (Figure 7C), the long exon that starts from chr2: 218,392,114 ends at chr2: 218,393,130 was skipped. MPP children had more A3SS spliced transcript events of SLC11A1 comparing to control children, which may explain the downregulation of SLC11A1 in MPP children.

## Discussion

There are still many difficulties in the effective treatment of children MPP. This study is to explore the immune-pathogenesis behind the acute lung injuries of MPP children and provide insight into the factors which may be helpful to the effective treatment of children MPP.

Transcriptome of BALF was analyzed with next-generation sequencing, 412 upregulated genes were identified in MPP group comparing to control group. Upregulated RLTPR, CARD11, and RASAL3 were significantly enriched to the GO terms of positive regulation of mononuclear cell proliferation and signaling in MPP children. RLTPR acts as a scaffold to bridge CD28 to CARD11 cytosolic adaptor and stimulate T cells (22, 23). RASAL3 is involved in the expansion and functions of liver NKT cells and the maintenance of optimal peripheral naive T cell numbers *in vivo* (24, 25). MPP has been characterized by the acute accumulation of mononuclear cells along the respiratory airways (26, 27). At the later stages of MPP, further destruction of airway parenchyma becomes obvious as a result of massive T cell infiltration (28). Therefore, we deduce that the upregulation of RLTPR, CARD11 and RASAL3 could increase the proliferation of the mononuclear cells in MPP children.

Kyoto encyclopedia of genes and genomes analysis found that hematopoietic cell linage pathway and T cell receptor signaling pathway were significantly upregulated in BALF of MPP children. After infection, MP antigen may activate CD8+ T cells through TCR; upregulated RLTPR and CARD11 genes may co-stimulate CD8+ T cells through CD28. Subsequently, activated CD8+ T cells highly produce IFNγ, which may be assisted by upregulated RASAL3. We found increased differentiation and proliferation of CD8+ T cells in BALF from MPP children, similar changes were also reported in MP-infected mice (29). CD8+ T cells can modulate immune and inflammatory responses against infectious agents through the production of IFNγ (29), which activate NK or macrophage cells (28). In addition, CD8+ T cells directly kill intracellular bacteria by producing substances including granulysin (GNLY) (30–32); CD4+ T cells may assist this process (33). Study on the lung lesions in goats revealed that the activation of CD4+ T lymphocytes plays a prominent role in the acute phase of the infection (34). MP infected laboratory rodents also showed high concentrations of CD4+ T cells within the inflammatory sites (35). However, no significant changes of the CD4 levels were found in this study of children BALF samples. It will be interesting to confirm and elucidate these different findings between animal and human samples.

Furthermore, we have provided evidence to support that innate immune responses are involved in children MPP. NK cells usually initiate immune responses against microbial infection (36). NK cells are reported to play an important role in the initial phase of *Mycoplasma* infection in mice (37). However, to the best of our knowledge, no report of NK cell expression in the BALF of children MPP comparing to FB control has been published yet. The specific NK cell pathways involved in the pathogenesis of children MPP remain to be clarified. Based on KEGG analysis, we found that NK cells were significantly activated, downstream genes were upregulated, and finally, the secretion of IFNγ was increased in MPP children. In addition, NK-originated IFNγ may activate IFNγR on target cells, induce their FAS expression and finally induce their apoptosis. Consistently, literature reported that NK-originated IFNγ was critical in dampening disease pathology and *Mycoplasma* growth in MPP of BALB/c mice (38). NK-originated IFNγ is critical in controlling *Mycoplasma* disease in early infection in mice (37).

Natural killer cells kill target cells in two major pathways (39). The first is the granule-exocytosis pathway, perforin and granzymes secreted by exocytosis activate cell-death mechanisms with or without the activation of caspases. The second pathway involves the engagement of FAS/CD95 on target cells and their cognate ligands FASL on NK cells, results in classical caspase-dependent apoptosis. Perforin is a membrane-disrupting protein that allows delivery of the proapoptotic granzymes into the cytosol of the target cells (40, 41). NK-originated perforin and granzyme may be mobilized by functional serine–threonine mitogen-activated protein kinase family members and finally induce the apoptosis of target cells (42–45). This study found that perforin, granzyme, and FASL were significantly upregulated in BALF of MPP children. We hypothesize that after MP infection, activated NK cells produce large amount of perforin, granzyme, and FASL, which kill both MP pathogens and target host cells. As a defense process of the host immune system, it may also play an important role in the formation of acute lung injury at the same time.

Alternative splicing of genes contributes to the physiological regulation of various biological systems (46), dysregulation of alternative splicing is often linked to various human diseases (47). GNLY and SLC11A1 genes were identified to have significant alternative splicing between MPP children and control children, which may cause the upregulation of GNLY gene and the downregulation of SLC11A1 in MPP children. GNLY is a cytolytic protein that presents in the granules of activated human NK cells (48). According to literature and our PPI analysis of granzyme (45), GNLY may be involved in the cytotoxic effects of NK cells in MPP. SLC11A1 is expressed exclusively in macrophages (49), the downregulation of SLC11A1 may be involved in the pathogenesis of MPP by affecting the function of macrophages (50).

There are some limitations in this study. First, this is a transcriptome analysis between six MPP children and three control children, bigger sized analysis would be preferred to support solid conclusions. However, each sample has been chosen carefully to receive next-generation sequencing separately; quality control analysis has been greatly satisfied; correlation matrix shows a high consistency of measurements within each group; PCA shows good repeatability of these samples. In addition, the upregulation of each gene involved in the KEGG pathways has been confirmed by qRT-PCR method in expanded patient groups, which may help to solid our findings. Second, the upregulated IFNγ has been found to be a very important cytokine in the KEGG enrichment analysis; however, the origin of IFNγ has not been clarified. IFNγ may be secreted by NK cells or T cells in BALF of MPP children. Further studies are required to clarify the exact origin of IFNγ and its specific pathogenesis role in MPP.

In conclusion, this study presents novel gene expression profiles as well as alternative splicing in BALF from MPP children by next-generation RNA sequencing. Mononuclear cell proliferation and signaling related genes including RLTPR, CARD11, and RASAL3 have been significantly upregulated in MPP children comparing to control. Furthermore, KEGG pathway analysis reveals that NK cell-mediated cytotoxicity pathway and T cell receptor signaling pathway have been significantly activated, which indicates that the activation of NK and CD8+ T cells may be indispensable in the pathogenesis of children MPP. In addition, the differential expression of GNLY and SLC11A1 in MPP children may be due to alternative splicing, further studies will be required to confirm this hypothesis.

## Ethics Statement

The study was approved by the Institutional Medical Ethics Review Board of the First Hospital of Jilin University in compliance with the Declaration of Helsinki; the reference number was 2015-238. The written informed consents were obtained by care givers of all children.

## Author Contributions

MG determined the clinical status for each children involved in the study, contributed to the interpretation of the data, and drafted the manuscript; KW performed experiments, contributed to the interpretation of data, and made the figures and tables; MY performed statistical analysis, helped with experiments, figures, and tables; FM participated in study design, coordination, and data interpretation; RL and HZ provided the samples, collected and interpreted the clinical information; GC participated in study design and data analysis; XW designed the study, analyzed data, and finished and revised the manuscript; all of the authors read and approved the manuscript.

## Conflict of Interest Statement

The authors declare that the research was conducted in the absence of any commercial or financial relationships that could be construed as a potential conflict of interest.

## Supplementary Material

Figure S1 Protein interaction network analysis. The first neighbors of CD8A **(A)**, granzyme B **(B)**, and IFNG **(C)** were decided based on Search Tool for the Retrieval of Interacting Genes/Proteins database. The color of each dot reflects the log2 fold change of each gene, the red dots represent upregulated proteins, and the green dots represent downregulated protein. The proteins in big and blue font are associated with the specific Kyoto encyclopedia of genes and genomes pathways mentioned in Figures 3–5.

Figure S2 Other genes mapped to hematopoietic cell lineage Kyoto encyclopedia of genes and genomes pathway. Model diagram shows hematopoietic cell lineage pathway, which included the differentiation of mast cells, basophils, eosinophils, myeloid-related dendritic cells, macrophages, and neutrophils. The green squares represent genes that did not have significant differences between *Mycoplasma pneumoniae* pneumonia group and foreign body group. The white squares represent genes that were not detectable.

## Abbreviations

A3SS, alternative 3′ splice sites; BAL, bronchoalveolar lavage; BALF, bronchoalveolar lavage fluid; CARD11, caspase recruitment domain family member 11; CAP, community-acquired pneumonia; CRP, c-reaction protein; CTL, c-type lectin; DEG, differential expression gene; DNA, deoxyribonucleic acid; FASL, Fas ligand; FB, foreign body; FDR, false discovery rate; FPKM, fragments per kilobase of transcript per million mapped fragments; GAPDH, glyceraldehyde-3-phosphate dehydrogenase; GNLY, granulysin; GO, gene ontology; GZMB, granzyme B; IFNG/IFNγ, interferon gamma; IGV, integrative genomics viewer; KEGG, Kyoto encyclopedia of genes and genomes; LG, *Legionella pneumophila*; MP, *Mycoplasma pneumoniae*; MPP, *Mycoplasma pneumoniae* pneumonia; NK, natural killer; PCR, polymerase chain reaction; PRF1, perforin 1 (pore forming protein); qRT-PCR, quantitative real-time PCR; RASAL3, RAS protein activator like 3; RI, retained intron; RLTPR, RGD motif, leucine rich repeats, tropomodulin domain and proline-rich containing; RNA, ribonucleic acid; SLC11A1, solute carrier family 11; STRING, Search Tool for the Retrieval of Interacting Genes/Proteins; Th1, T helper type 1; WBC, white blood cell.
