# Supplementary figures and images for: Transcriptome Analysis of Bronchoalveolar Lavage Fluid From Children With Mycoplasma pneumoniae Pneumonia Reveals Natural Killer and T Cell-Proliferation Responses
Source: Front Immunol. 2018 Jun 18;9:1403. doi: 10.3389/fimmu.2018.01403 (PMC6015898; doi:10.3389/fimmu.2018.01403)

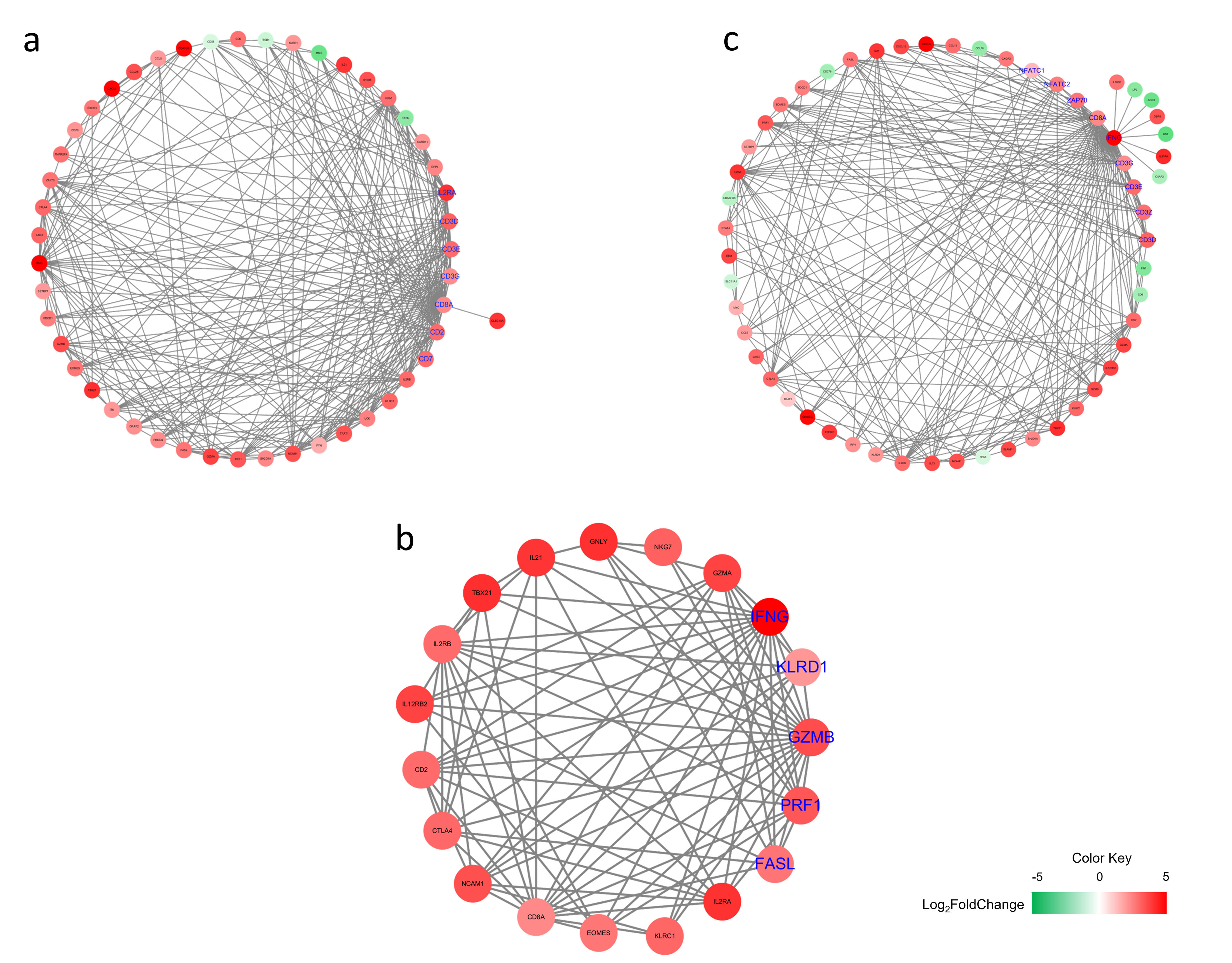

Supplement: Figure S1 — Protein interaction network analysis. The first neighbors of CD8A (A), granzyme B (B), and IFNG (C) were decided based on Search Tool for the Retrieval of Interacting Genes/Proteins database. The color of each dot reflects the log2 fold change of each gene, the red dots represent upregulated proteins, and the green dots represent downregulated protein. The proteins in big and blue font are associated with the specific Kyoto encyclopedia of genes and genomes pathways mentioned in Figures 3–5. [file image_1.tif]

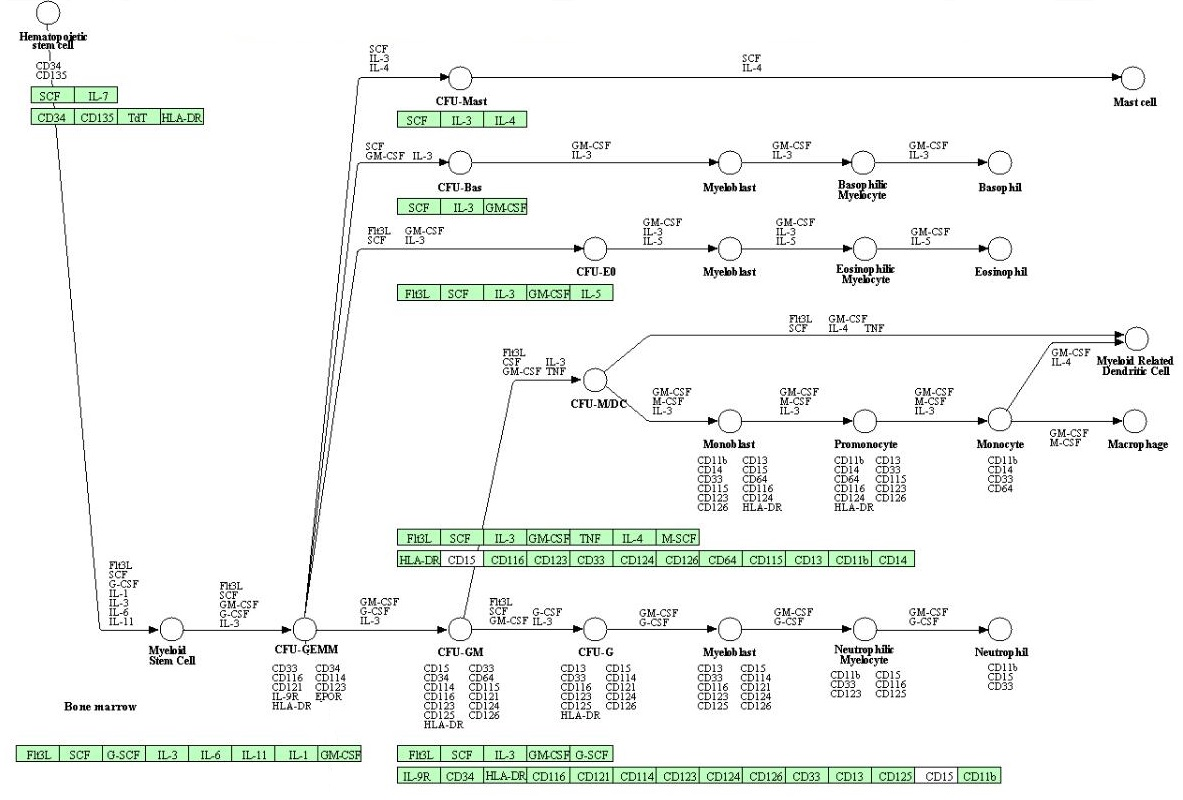

Supplement: Figure S2 — Other genes mapped to hematopoietic cell lineage Kyoto encyclopedia of genes and genomes pathway. Model diagram shows hematopoietic cell lineage pathway, which included the differentiation of mast cells, basophils, eosinophils, myeloid-related dendritic cells, macrophages, and neutrophils. The green squares represent genes that did not have significant differences between Mycoplasma pneumoniae pneumonia group and foreign body group. The white squares represent genes that were not detectable. [file image_2.tif]
