# Supplementary material for: Transcriptome Analysis of Bronchoalveolar Lavage Fluid From Children With Mycoplasma pneumoniae Pneumonia Reveals Natural Killer and T Cell-Proliferation Responses
Source: Front Immunol. 2018 Jun 18;9:1403. doi: 10.3389/fimmu.2018.01403 (PMC6015898; doi:10.3389/fimmu.2018.01403)
Supplement: Supplementary file 3 [file table_1.doc]

**Additional File 1: Table S1. Summary of patients’ information.**

| Sample number | Control 1 | Control 2 | Control 3 | MPP 1 | MPP 2 | MPP 3 | MPP 4 | MPP 5 | MPP 6 |  |
| --- | --- | --- | --- | --- | --- | --- | --- | --- | --- | --- |
| Age (year) | 1.4 | 1.8 | 2.1 | 13 | 6 | 7 | 4 | 6 | 2 |  |
| Sex (M/F) | F | M | M | F | M | F | F | M | M |  |
| Diagnosis | FB | FB | FB | MPP | MPP | MPP | MPP | MPP | MPP |  |
| Hospitalization days | < 24h | < 24h | < 24h | 7 | 12 | 4 | 13 | 10 | 17 |  |
| Body temperature (℃) | 36.5 | 36.8 | 36.7 | 37.5 | 38.3 | 38 | 40 | 39.5 | 40.3 |  |
| Respiratory rate (breaths/min) | 35 | 30 | 28 | 20 | 25 | 25 | 55 | 50 | 70 |  |
| Nasal flaring | NO | NO | NO | NO | NO | NO | YES | YES | YES |  |
| Retractions | NO | NO | NO | NO | NO | NO | YES | YES | YES |  |
| Cyanosis | NO | NO | NO | NO | NO | NO | YES | YES | YES |  |
| Fever days | NO | NO | NO | 3 | 9 | 5 | 11 | 9 | 14 |  |
| WBC (×10^10/L) | 6.31 | 7.25 | 4.68 | 7.17 | 5.35 | 6.18 | 8.37 | 6.48 | 8.64 |  |
| NE% | 45 | 53 | 59 | 51 | 70 | 55 | 68 | 79 | 55 |  |
| LY% | 44 | 38 | 33 | 36 | 17 | 34 | 18 | 13 | 35 |  |
| MO% | 10 | 8 | 6 | 8 | 9 | 9 | 8 | 5 | 9 |  |
| CRP (mg/L) | 3 | 0.5 | 5 | 10 | 33.9 | 23.7 | 29.9 | 14.7 | 9.52 |  |
| MP-Ab | Negative | Negative | Negative | 1:40 | 1:80 | 1:40 | 1:320 | 1:320 | 1:320 |  |
| Pleural effusion | NO | NO | NO | NO | NO | NO | NO | YES | YES |  |
| General/ Refractory | - | - | - | General | General | General | Refractory | Refractory | Refractory |  |
| Glucocorticoids | NO | NO | NO | NO | NO | NO | YES | YES | YES |  |
| FB: Foreign Body Aspiration, BALF for control was collected at the time of re-examination of bronchus foreign body; MPP: *Mycoplasma pneumoniae* Pneumonia, BALF for MPP was collected within one week after onset of the pneumonia. | | | | | | | | | | |
