# Supplementary material for: Transcriptome Analysis of Bronchoalveolar Lavage Fluid From Children With Mycoplasma pneumoniae Pneumonia Reveals Natural Killer and T Cell-Proliferation Responses
Source: Front Immunol. 2018 Jun 18;9:1403. doi: 10.3389/fimmu.2018.01403 (PMC6015898; doi:10.3389/fimmu.2018.01403)
Supplement: Supplementary file 4 [file table_2.doc]

| **Additional file 2: Table S2.** Lymphocyte profiles in BALF of MPP children and control children | | | | | | | | | | | |
| --- | --- | --- | --- | --- | --- | --- | --- | --- | --- | --- | --- |
| Samples | Total CD3+ T Cells (cells/μl) | Total CD3+ T Cells (%) | CD3+CD4+ T Cells (cells/μl) | CD3+CD4+ T Cells (%) | CD3+CD8+ T Cells (cells/μl) | CD3+CD8+ T Cells (%) | CD4+/  CD8+ | CD19+ B Cells (cells/μl) | CD19+ B Cells (%) | D16+CD56+ NK Cells (cells/μl) | CD16+CD56+ NK Cells (%) |
| Control (n = 5) | 114.18±29.73 | 77.04±12 | 40.57±17.98 | 28.55±8.87 | 39.27±22.75 | 25.75±7.88 | 1.15±0.26 | 2.35±1.11 | 1.46±0.23 | 34.1±27.97 | 19.03±10.51 |
| MPP  (n = 10) | 834.77±566.12 | 80.92±9.45 | 440.77±377.41 | 39.72±15.69 | 303.88±167.46 | 33.4±10.96 | 1.36±0.78 | 21.18±13.3 | 2.53±1.83 | 126.77±88.14 | 14.51±9.36 |
| *p* value | 0.0007^***^ | 0.7679 | 0.0047^**^ | 0.3097 | 0.0007^***^ | 0.1775 | 0.7679 | 0.0007^***^ | 0.5135 | 0.0280^*^ | 0.5941 |
| * *p* < 0.05, ** *p* < 0.01, *** *p* < 0.001 | | | | | | | | | | | |
