# Supplementary material for: Transcriptome Analysis of Bronchoalveolar Lavage Fluid From Children With Mycoplasma pneumoniae Pneumonia Reveals Natural Killer and T Cell-Proliferation Responses
Source: Front Immunol. 2018 Jun 18;9:1403. doi: 10.3389/fimmu.2018.01403 (PMC6015898; doi:10.3389/fimmu.2018.01403)
Supplement: Supplementary file 5 [file table_3.doc]

| **Additional file 3: Table S3. Nucleated cell count in the BALF samples** | | | | | |
| --- | --- | --- | --- | --- | --- |
| Samples | Lymphocyte (%) | Macrophages (%) | Neutrophils (%) | Eosinophils (%) | Nucleated cell count（10^6/L） |
| Control 1 | 8 | 87 | 2 | 0 | 994 |
| Control 2 | 9 | 90 | 1 | 0 | 1174 |
| Control 3 | 16 | 78 | 6 | 0 | 1150 |
| MPP 1 | 26 | 66 | 5 | 0.000 | 5025 |
| MPP 2 | 28 | 50 | 4 | 0.000 | 2969 |
| MPP 3 | 19 | 76 | 2 | 0.000 | 2952 |
| MPP 4 | 6 | 92 | 2 | 0.000 | 3607 |
| MPP 5 | 33 | 35 | 25 | 0.000 | 5750 |
| MPP 6 | 29 | 61 | 3 | 1.000 | 5699 |
| *p* value (Control vs MPP) | 0.1667 | 0.1667 | 0.548 | - | 0.0238 |
