# Supplementary material for: Transcriptome Analysis of Bronchoalveolar Lavage Fluid From Children With Mycoplasma pneumoniae Pneumonia Reveals Natural Killer and T Cell-Proliferation Responses
Source: Front Immunol. 2018 Jun 18;9:1403. doi: 10.3389/fimmu.2018.01403 (PMC6015898; doi:10.3389/fimmu.2018.01403)
Supplement: Supplementary file 6 [file table_4.doc]

**Additional File 4: Table S4. Summary of the RNA sequencing reads and their mapping results.**

| Sample name | Control 1 | Control 2 | Control 3 | MPP 1 | MPP 2 | MPP 3 | MPP 4 | MPP 5 | MPP 6 |
| --- | --- | --- | --- | --- | --- | --- | --- | --- | --- |
| Total reads | 70791680 | 43660754 | 41348750 | 55492664 | 47186542 | 64792680 | 68897254 | 42136856 | 46318894 |
| Total mapped | 61895353 (87.43%) | 38130879 (87.33%) | 35794581 (86.57%) | 48535137 (87.46%) | 40517410 (85.87%) | 56013052 (86.45%) | 60481397 (87.78%) | 36565361 (86.78%) | 40224088 (86.84%) |
| Multiple mapped | 925547 (1.31%) | 538865 (1.23%) | 489708 (1.18%) | 844736 (1.52%) | 614681 (1.3%) | 797662 (1.23%) | 881129 (1.28%) | 543196 (1.29%) | 630869 (1.36%) |
| Uniquely mapped | 60969806 (86.13%) | 37592014 (86.1%) | 35304873 (85.38%) | 47690401 (85.94%) | 39902729 (84.56%) | 55215390 (85.22%) | 59600268 (86.51%) | 36022165 (85.49%) | 39593219 (85.48%) |
| Reads map to '+' | 30481040 (43.06%) | 18792913 (43.04%) | 17657164 (42.7%) | 23844413 (42.97%) | 19967766 (42.32%) | 27594117 (42.59%) | 29790936 (43.24%) | 18008541 (42.74%) | 19797319 (42.74%) |
| Reads map to '-' | 30488766 (43.07%) | 18799101 (43.06%) | 17647709 (42.68%) | 23845988 (42.97%) | 19934963 (42.25%) | 27621273 (42.63%) | 29809332 (43.27%) | 18013624 (42.75%) | 19795900 (42.74%) |
| Non-splice reads | 40139644 (56.7%) | 22376959 (51.25%) | 20308056 (49.11%) | 30580304 (55.11%) | 22475106 (47.63%) | 34530918 (53.29%) | 37577003 (54.54%) | 20733762 (49.21%) | 24205574 (52.26%) |
| Splice reads | 20830162 (29.42%) | 15215055 (34.85%) | 14996817 (36.27%) | 17110097 (30.83%) | 17427623 (36.93%) | 20684472 (31.92%) | 22023265 (31.97%) | 15288403 (36.28%) | 15387645 (33.22%) |

Total reads: The number of sequence after sequencing data filtering.

Total mapped: The number of sequence which can map to the genome.

Multiple mapped: The number of sequence which have multiple location on the reference sequencing.

Uniquely mapped: The number of sequence which have Single location on the reference sequencing.

Reads map to ‘+’: The statistics of sequence that were mapped on the ‘+’chain of the genome.

Reads map to ‘-’: The statistics of sequence that were mapped on the ‘-’chain of the genome.
