# Supplementary material for: Transcriptome Analysis of Bronchoalveolar Lavage Fluid From Children With Mycoplasma pneumoniae Pneumonia Reveals Natural Killer and T Cell-Proliferation Responses
Source: Front Immunol. 2018 Jun 18;9:1403. doi: 10.3389/fimmu.2018.01403 (PMC6015898; doi:10.3389/fimmu.2018.01403)
Supplement: Supplementary file 7 [file table_5.doc]

| Gene_ID  **Additional File 5: Table S5. Significantly up-regulated genes of MPP group vs control group.** | Gene Name | log_2_FoldChange | padj | Description |
| --- | --- | --- | --- | --- |
| ENSG00000073861 | TBX21 | 2.7251 | 7.11E-18 | T-box 21 [Source:HGNC Symbol;Acc:HGNC:11599] |
| ENSG00000102245 | CD40LG | 3.2455 | 5.48E-12 | CD40 ligand [Source:HGNC Symbol;Acc:HGNC:11935] |
| ENSG00000177494 | ZBED2 | 3.0813 | 1.75E-07 | zinc finger, BED-type containing 2 [Source:HGNC Symbol;Acc:HGNC:20710] |
| ENSG00000188389 | PDCD1 | 1.706 | 1.75E-07 | programmed cell death 1 [Source:HGNC Symbol;Acc:HGNC:8760] |
| ENSG00000103522 | IL21R | 1.9672 | 2.58E-07 | interleukin 21 receptor [Source:HGNC Symbol;Acc:HGNC:6006] |
| ENSG00000116824 | CD2 | 1.9473 | 1.10E-06 | CD2 molecule [Source:HGNC Symbol;Acc:HGNC:1639] |
| ENSG00000008517 | IL32 | 2.2363 | 1.34E-06 | interleukin 32 [Source:HGNC Symbol;Acc:HGNC:16830] |
| ENSG00000235576 | AC092580.4 | 2.2898 | 3.20E-06 | - |
| ENSG00000167286 | CD3D | 2.0628 | 3.23E-06 | CD3d molecule, delta (CD3-TCR complex) [Source:HGNC Symbol;Acc:HGNC:1673] |
| ENSG00000104490 | NCALD | 2.1535 | 4.42E-06 | neurocalcin delta [Source:HGNC Symbol;Acc:HGNC:7655] |
| ENSG00000081985 | IL12RB2 | 2.4767 | 1.65E-05 | interleukin 12 receptor subunit beta 2 [Source:HGNC Symbol;Acc:HGNC:5972] |
| ENSG00000186827 | TNFRSF4 | 1.8671 | 1.93E-05 | tumor necrosis factor receptor superfamily member 4 [Source:HGNC Symbol;Acc:HGNC:11918] |
| ENSG00000255833 | TIFAB | 2.441 | 1.94E-05 | TRAF-interacting protein with forkhead-associated domain, family member B [Source:HGNC Symbol;Acc:HGNC:34024] |
| ENSG00000100385 | IL2RB | 1.9423 | 2.09E-05 | interleukin 2 receptor subunit beta [Source:HGNC Symbol;Acc:HGNC:6009] |
| ENSG00000198821 | CD247 | 1.8956 | 3.62E-05 | CD247 molecule [Source:HGNC Symbol;Acc:HGNC:1677] |
| ENSG00000162676 | GFI1 | 1.4147 | 5.26E-05 | growth factor independent 1 transcription repressor [Source:HGNC Symbol;Acc:HGNC:4237] |
| ENSG00000181036 | FCRL6 | 1.952 | 5.26E-05 | Fc receptor-like 6 [Source:HGNC Symbol;Acc:HGNC:31910] |
| ENSG00000137441 | FGFBP2 | 2.8162 | 6.13E-05 | fibroblast growth factor binding protein 2 [Source:HGNC Symbol;Acc:HGNC:29451] |
| ENSG00000111537 | IFNG | 3.3439 | 6.26E-05 | interferon, gamma [Source:HGNC Symbol;Acc:HGNC:5438] |
| ENSG00000243772 | KIR2DL3 | 2.6912 | 6.88E-05 | killer cell immunoglobulin-like receptor, two domains, long cytoplasmic tail, 3 [Source:HGNC Symbol;Acc:HGNC:6331] |
| ENSG00000233093 | LINC00892 | 2.7731 | 7.55E-05 | long intergenic non-protein coding RNA 892 [Source:HGNC Symbol;Acc:HGNC:48578] |
| ENSG00000198885 | ITPRIPL1 | 1.7599 | 8.67E-05 | inositol 1,4,5-trisphosphate receptor interacting protein-like 1 [Source:HGNC Symbol;Acc:HGNC:29371] |
| ENSG00000124785 | NRN1 | 2.7769 | 0.00010058 | neuritin 1 [Source:HGNC Symbol;Acc:HGNC:17972] |
| ENSG00000157303 | SUSD3 | 1.4644 | 0.00010525 | sushi domain containing 3 [Source:HGNC Symbol;Acc:HGNC:28391] |
| ENSG00000139737 | SLAIN1 | 1.7132 | 0.00012141 | SLAIN motif family member 1 [Source:HGNC Symbol;Acc:HGNC:26387] |
| ENSG00000240350 | AC017002.1 | 2.5727 | 0.00013068 | - |
| ENSG00000140284 | SLC27A2 | 2.234 | 0.00013958 | solute carrier family 27 (fatty acid transporter), member 2 [Source:HGNC Symbol;Acc:HGNC:10996] |
| ENSG00000211776 | TRAV2 | 1.9805 | 0.0001551 | T cell receptor alpha variable 2 [Source:HGNC Symbol;Acc:HGNC:12116] |
| ENSG00000100162 | CENPM | 1.8002 | 0.00015546 | centromere protein M [Source:HGNC Symbol;Acc:HGNC:18352] |
| ENSG00000132514 | CLEC10A | 2.6629 | 0.0001569 | C-type lectin domain family 10 member A [Source:HGNC Symbol;Acc:HGNC:16916] |
| ENSG00000166105 | GLB1L3 | 2.534 | 0.00015959 | galactosidase beta 1 like 3 [Source:HGNC Symbol;Acc:HGNC:25147] |
| ENSG00000174944 | P2RY14 | 2.7187 | 0.00017239 | purinergic receptor P2Y, G-protein coupled, 14 [Source:HGNC Symbol;Acc:HGNC:16442] |
| ENSG00000101096 | NFATC2 | 1.7124 | 0.00018554 | nuclear factor of activated T-cells, cytoplasmic, calcineurin-dependent 2 [Source:HGNC Symbol;Acc:HGNC:7776] |
| ENSG00000138755 | CXCL9 | 3.3051 | 0.00019476 | chemokine (C-X-C motif) ligand 9 [Source:HGNC Symbol;Acc:HGNC:7098] |
| ENSG00000173762 | CD7 | 1.9497 | 0.00020165 | CD7 molecule [Source:HGNC Symbol;Acc:HGNC:1695] |
| ENSG00000089012 | SIRPG | 2.417 | 0.00020747 | signal-regulatory protein gamma [Source:HGNC Symbol;Acc:HGNC:15757] |
| ENSG00000027869 | SH2D2A | 1.9458 | 0.00022924 | SH2 domain containing 2A [Source:HGNC Symbol;Acc:HGNC:10821] |
| ENSG00000105246 | EBI3 | 2.4457 | 0.00024679 | Epstein-Barr virus induced 3 [Source:HGNC Symbol;Acc:HGNC:3129] |
| ENSG00000137265 | IRF4 | 1.4392 | 0.00024679 | interferon regulatory factor 4 [Source:HGNC Symbol;Acc:HGNC:6119] |
| ENSG00000101445 | PPP1R16B | 1.7992 | 0.00025119 | protein phosphatase 1 regulatory subunit 16B [Source:HGNC Symbol;Acc:HGNC:15850] |
| ENSG00000105374 | NKG7 | 2.0752 | 0.00025765 | natural killer cell granule protein 7 [Source:HGNC Symbol;Acc:HGNC:7830] |
| ENSG00000115523 | GNLY | 2.7199 | 0.00034523 | granulysin [Source:HGNC Symbol;Acc:HGNC:4414] |
| ENSG00000169583 | CLIC3 | 1.5988 | 0.00037956 | chloride intracellular channel 3 [Source:HGNC Symbol;Acc:HGNC:2064] |
| ENSG00000135426 | TESPA1 | 1.636 | 0.0003983 | thymocyte expressed, positive selection associated 1 [Source:HGNC Symbol;Acc:HGNC:29109] |
| ENSG00000186891 | TNFRSF18 | 1.7516 | 0.00041079 | tumor necrosis factor receptor superfamily member 18 [Source:HGNC Symbol;Acc:HGNC:11914] |
| ENSG00000105366 | SIGLEC8 | 3.2722 | 0.00042465 | sialic acid binding Ig-like lectin 8 [Source:HGNC Symbol;Acc:HGNC:10877] |
| ENSG00000143333 | RGS16 | 2.0397 | 0.00043375 | regulator of G-protein signaling 16 [Source:HGNC Symbol;Acc:HGNC:9997] |
| ENSG00000171476 | HOPX | 1.8931 | 0.00046383 | HOP homeobox [Source:HGNC Symbol;Acc:HGNC:24961] |
| Novel00050 | -- | 2.4515 | 0.00046399 | hCG1991981 [Homo sapiens] |
| ENSG00000134594 | RAB33A | 2.153 | 0.00047712 | RAB33A, member RAS oncogene family [Source:HGNC Symbol;Acc:HGNC:9773] |
| ENSG00000135736 | CCDC102A | 2.005 | 0.00051819 | coiled-coil domain containing 102A [Source:HGNC Symbol;Acc:HGNC:28097] |
| ENSG00000138134 | STAMBPL1 | 1.5972 | 0.00055167 | STAM binding protein like 1 [Source:HGNC Symbol;Acc:HGNC:24105] |
| ENSG00000243811 | APOBEC3D | 1.4731 | 0.00056629 | apolipoprotein B mRNA editing enzyme, catalytic polypeptide-like 3D [Source:HGNC Symbol;Acc:HGNC:17354] |
| ENSG00000211772 | TRBC2 | 1.9125 | 0.00057747 | T cell receptor beta constant 2 [Source:HGNC Symbol;Acc:HGNC:12157] |
| ENSG00000198851 | CD3E | 1.9376 | 0.00058327 | CD3e molecule, epsilon (CD3-TCR complex) [Source:HGNC Symbol;Acc:HGNC:1674] |
| ENSG00000163519 | TRAT1 | 2.2439 | 0.00059801 | T cell receptor associated transmembrane adaptor 1 [Source:HGNC Symbol;Acc:HGNC:30698] |
| ENSG00000146670 | CDCA5 | 1.8947 | 0.00066888 | cell division cycle associated 5 [Source:HGNC Symbol;Acc:HGNC:14626] |
| ENSG00000273604 | C17orf96 | 2.3675 | 0.00066888 | chromosome 17 open reading frame 96 [Source:HGNC Symbol;Acc:HGNC:34493] |
| ENSG00000211716 | TRBV9 | 2.7847 | 0.00067437 | T cell receptor beta variable 9 [Source:HGNC Symbol;Acc:HGNC:12246] |
| ENSG00000078596 | ITM2A | 1.9492 | 0.00068351 | integral membrane protein 2A [Source:HGNC Symbol;Acc:HGNC:6173] |
| ENSG00000115085 | ZAP70 | 1.8409 | 0.00071222 | zeta chain of T cell receptor associated protein kinase 70kDa [Source:HGNC Symbol;Acc:HGNC:12858] |
| ENSG00000101082 | SLA2 | 1.6169 | 0.00092346 | Src-like-adaptor 2 [Source:HGNC Symbol;Acc:HGNC:17329] |
| ENSG00000011590 | ZBTB32 | 1.3127 | 0.00099415 | zinc finger and BTB domain containing 32 [Source:HGNC Symbol;Acc:HGNC:16763] |
| ENSG00000225720 | RP4-742C19.12 | 2.601 | 0.0010197 | - |
| ENSG00000133321 | RARRES3 | 1.9973 | 0.0010245 | retinoic acid receptor responder (tazarotene induced) 3 [Source:HGNC Symbol;Acc:HGNC:9869] |
| ENSG00000137078 | SIT1 | 2.1015 | 0.0010245 | signaling threshold regulating transmembrane adaptor 1 [Source:HGNC Symbol;Acc:HGNC:17710] |
| ENSG00000172551 | MUCL1 | 3.3507 | 0.0010308 | mucin-like 1 [Source:HGNC Symbol;Acc:HGNC:30588] |
| ENSG00000101057 | MYBL2 | 2.1298 | 0.0010579 | v-myb avian myeloblastosis viral oncogene homolog-like 2 [Source:HGNC Symbol;Acc:HGNC:7548] |
| ENSG00000119403 | PHF19 | 1.037 | 0.0011589 | PHD finger protein 19 [Source:HGNC Symbol;Acc:HGNC:24566] |
| ENSG00000152969 | JAKMIP1 | 2.0148 | 0.0011608 | janus kinase and microtubule interacting protein 1 [Source:HGNC Symbol;Acc:HGNC:26460] |
| ENSG00000113263 | ITK | 1.3708 | 0.0012946 | IL2-inducible T-cell kinase [Source:HGNC Symbol;Acc:HGNC:6171] |
| ENSG00000183337 | BCOR | 1.1558 | 0.0012946 | BCL6 corepressor [Source:HGNC Symbol;Acc:HGNC:20893] |
| ENSG00000228672 | PROB1 | 1.5817 | 0.0012946 | proline-rich basic protein 1 [Source:HGNC Symbol;Acc:HGNC:41906] |
| ENSG00000116661 | FBXO2 | 2.0694 | 0.001328 | F-box protein 2 [Source:HGNC Symbol;Acc:HGNC:13581] |
| ENSG00000116774 | OLFML3 | 2.0742 | 0.0014201 | olfactomedin like 3 [Source:HGNC Symbol;Acc:HGNC:24956] |
| ENSG00000213626 | LBH | 1.6473 | 0.0014369 | limb bud and heart development [Source:HGNC Symbol;Acc:HGNC:29532] |
| ENSG00000154839 | SKA1 | 1.7819 | 0.0014507 | spindle and kinetochore associated complex subunit 1 [Source:HGNC Symbol;Acc:HGNC:28109] |
| ENSG00000235505 | RP11-693N9.2 | 1.7332 | 0.0014784 | - |
| ENSG00000198286 | CARD11 | 1.4338 | 0.0014891 | caspase recruitment domain family member 11 [Source:HGNC Symbol;Acc:HGNC:16393] |
| ENSG00000159618 | ADGRG5 | 1.9299 | 0.0015026 | adhesion G protein-coupled receptor G5 [Source:HGNC Symbol;Acc:HGNC:19010] |
| ENSG00000206561 | COLQ | 2.2135 | 0.0016083 | collagen-like tail subunit (single strand of homotrimer) of asymmetric acetylcholinesterase [Source:HGNC Symbol;Acc:HGNC:2226] |
| ENSG00000149150 | SLC43A1 | 1.6442 | 0.0017395 | solute carrier family 43 (amino acid system L transporter), member 1 [Source:HGNC Symbol;Acc:HGNC:9225] |
| ENSG00000091972 | CD200 | 1.9219 | 0.0017547 | CD200 molecule [Source:HGNC Symbol;Acc:HGNC:7203] |
| ENSG00000063180 | CA11 | 1.2979 | 0.0017768 | carbonic anhydrase XI [Source:HGNC Symbol;Acc:HGNC:1370] |
| ENSG00000134460 | IL2RA | 2.6895 | 0.0017768 | interleukin 2 receptor subunit alpha [Source:HGNC Symbol;Acc:HGNC:6008] |
| ENSG00000211721 | TRBV6-5 | 2.38 | 0.0017878 | T cell receptor beta variable 6-5 [Source:HGNC Symbol;Acc:HGNC:12230] |
| ENSG00000211899 | IGHM | 3.1528 | 0.0018067 | immunoglobulin heavy constant mu [Source:HGNC Symbol;Acc:HGNC:5541] |
| ENSG00000069702 | TGFBR3 | 1.5262 | 0.0019305 | transforming growth factor beta receptor III [Source:HGNC Symbol;Acc:HGNC:11774] |
| ENSG00000153283 | CD96 | 1.6903 | 0.0019305 | CD96 molecule [Source:HGNC Symbol;Acc:HGNC:16892] |
| ENSG00000113319 | RASGRF2 | 2.0324 | 0.0020157 | Ras protein specific guanine nucleotide releasing factor 2 [Source:HGNC Symbol;Acc:HGNC:9876] |
| ENSG00000145649 | GZMA | 2.483 | 0.0022498 | granzyme A [Source:HGNC Symbol;Acc:HGNC:4708] |
| ENSG00000093009 | CDC45 | 1.6745 | 0.0022928 | cell division cycle 45 [Source:HGNC Symbol;Acc:HGNC:1739] |
| ENSG00000154451 | GBP5 | 2.2562 | 0.0023101 | guanylate binding protein 5 [Source:HGNC Symbol;Acc:HGNC:19895] |
| ENSG00000168005 | C11orf84 | 0.84905 | 0.0023101 | chromosome 11 open reading frame 84 [Source:HGNC Symbol;Acc:HGNC:25115] |
| ENSG00000186174 | BCL9L | 1.2625 | 0.0023101 | B-cell CLL/lymphoma 9-like [Source:HGNC Symbol;Acc:HGNC:23688] |
| ENSG00000178999 | AURKB | 1.5183 | 0.0027441 | aurora kinase B [Source:HGNC Symbol;Acc:HGNC:11390] |
| ENSG00000222041 | LINC00152 | 1.1854 | 0.0028187 | long intergenic non-protein coding RNA 152 [Source:HGNC Symbol;Acc:HGNC:28717] |
| ENSG00000129521 | EGLN3 | 2.1229 | 0.0028199 | egl-9 family hypoxia-inducible factor 3 [Source:HGNC Symbol;Acc:HGNC:14661] |
| ENSG00000205683 | DPF3 | 1.682 | 0.0028199 | D4, zinc and double PHD fingers, family 3 [Source:HGNC Symbol;Acc:HGNC:17427] |
| ENSG00000083720 | OXCT1 | 1.3658 | 0.0028968 | 3-oxoacid CoA-transferase 1 [Source:HGNC Symbol;Acc:HGNC:8527] |
| ENSG00000100450 | GZMH | 2.2831 | 0.0029667 | granzyme H [Source:HGNC Symbol;Acc:HGNC:4710] |
| ENSG00000013725 | CD6 | 1.8561 | 0.0030819 | CD6 molecule [Source:HGNC Symbol;Acc:HGNC:1691] |
| ENSG00000168899 | VAMP5 | 2.2102 | 0.0031142 | vesicle associated membrane protein 5 [Source:HGNC Symbol;Acc:HGNC:12646] |
| ENSG00000182054 | IDH2 | 1.1367 | 0.0031492 | isocitrate dehydrogenase 2 (NADP+), mitochondrial [Source:HGNC Symbol;Acc:HGNC:5383] |
| ENSG00000155961 | RAB39B | 1.2072 | 0.003203 | RAB39B, member RAS oncogene family [Source:HGNC Symbol;Acc:HGNC:16499] |
| ENSG00000180096 | SEPTIN 1 | 1.8169 | 0.0033311 | septin 1 [Source:HGNC Symbol;Acc:HGNC:2879] |
| ENSG00000276231 | PIK3R6 | 1.6328 | 0.0036873 | phosphoinositide-3-kinase regulatory subunit 6 [Source:HGNC Symbol;Acc:HGNC:27101] |
| ENSG00000142185 | TRPM2 | 1.361 | 0.0037457 | transient receptor potential cation channel, subfamily M, member 2 [Source:HGNC Symbol;Acc:HGNC:12339] |
| ENSG00000153982 | GDPD1 | 1.5746 | 0.0037457 | glycerophosphodiester phosphodiesterase domain containing 1 [Source:HGNC Symbol;Acc:HGNC:20883] |
| ENSG00000137496 | IL18BP | 1.8513 | 0.0038153 | interleukin 18 binding protein [Source:HGNC Symbol;Acc:HGNC:5987] |
| ENSG00000117090 | SLAMF1 | 2.3831 | 0.0039626 | signaling lymphocytic activation molecule family member 1 [Source:HGNC Symbol;Acc:HGNC:10903] |
| ENSG00000133561 | GIMAP6 | 1.7152 | 0.0043099 | GTPase, IMAP family member 6 [Source:HGNC Symbol;Acc:HGNC:21918] |
| ENSG00000158555 | GDPD5 | 1.6458 | 0.0043198 | glycerophosphodiester phosphodiesterase domain containing 5 [Source:HGNC Symbol;Acc:HGNC:28804] |
| ENSG00000180644 | PRF1 | 2.2091 | 0.0043198 | perforin 1 (pore forming protein) [Source:HGNC Symbol;Acc:HGNC:9360] |
| ENSG00000169129 | AFAP1L2 | 2.0221 | 0.0043474 | actin filament associated protein 1 like 2 [Source:HGNC Symbol;Acc:HGNC:25901] |
| ENSG00000118971 | CCND2 | 1.3648 | 0.0044647 | cyclin D2 [Source:HGNC Symbol;Acc:HGNC:1583] |
| ENSG00000151136 | BTBD11 | 1.7817 | 0.0044647 | BTB (POZ) domain containing 11 [Source:HGNC Symbol;Acc:HGNC:23844] |
| ENSG00000188322 | SBK1 | 1.5554 | 0.0044647 | SH3 domain binding kinase 1 [Source:HGNC Symbol;Acc:HGNC:17699] |
| ENSG00000245954 | RP11-18H21.1 | 2.8083 | 0.004482 | - |
| ENSG00000280721 | AC133644.2 | 1.8099 | 0.0045567 | - |
| ENSG00000164509 | IL31RA | 2.8175 | 0.0045598 | interleukin 31 receptor A [Source:HGNC Symbol;Acc:HGNC:18969] |
| ENSG00000277734 | TRAC | 1.6787 | 0.0046287 | T-cell receptor alpha constant [Source:HGNC Symbol;Acc:HGNC:12029] |
| ENSG00000166803 | KIAA0101 | 1.6444 | 0.0046792 | KIAA0101 [Source:HGNC Symbol;Acc:HGNC:28961] |
| ENSG00000105376 | ICAM5 | 2.0195 | 0.0047116 | intercellular adhesion molecule 5 [Source:HGNC Symbol;Acc:HGNC:5348] |
| ENSG00000174946 | GPR171 | 1.453 | 0.0049819 | G protein-coupled receptor 171 [Source:HGNC Symbol;Acc:HGNC:30057] |
| ENSG00000154760 | SLFN13 | 1.0988 | 0.0050416 | schlafen family member 13 [Source:HGNC Symbol;Acc:HGNC:26481] |
| ENSG00000186810 | CXCR3 | 1.7862 | 0.0051633 | chemokine (C-X-C motif) receptor 3 [Source:HGNC Symbol;Acc:HGNC:4540] |
| ENSG00000211746 | TRBV19 | 2.0822 | 0.0052594 | T cell receptor beta variable 19 [Source:HGNC Symbol;Acc:HGNC:12194] |
| ENSG00000182162 | P2RY8 | 1.7681 | 0.0053505 | purinergic receptor P2Y, G-protein coupled, 8 [Source:HGNC Symbol;Acc:HGNC:15524] |
| ENSG00000197299 | BLM | 1.222 | 0.0053505 | Bloom syndrome, RecQ helicase-like [Source:HGNC Symbol;Acc:HGNC:1058] |
| ENSG00000130222 | GADD45G | 2.0405 | 0.0055028 | growth arrest and DNA damage inducible gamma [Source:HGNC Symbol;Acc:HGNC:4097] |
| ENSG00000229056 | AC020571.3 | 2.5002 | 0.0056105 | - |
| ENSG00000166886 | NAB2 | 1.1098 | 0.0056708 | NGFI-A binding protein 2 (EGR1 binding protein 2) [Source:HGNC Symbol;Acc:HGNC:7627] |
| ENSG00000225698 | IGHV3-72 | 3.0028 | 0.0058092 | immunoglobulin heavy variable 3-72 [Source:HGNC Symbol;Acc:HGNC:5622] |
| ENSG00000173040 | EVC2 | 1.1932 | 0.0058797 | Ellis van Creveld syndrome 2 [Source:HGNC Symbol;Acc:HGNC:19747] |
| ENSG00000168421 | RHOH | 1.8795 | 0.0059739 | ras homolog family member H [Source:HGNC Symbol;Acc:HGNC:686] |
| ENSG00000179144 | GIMAP7 | 1.7984 | 0.0059739 | GTPase, IMAP family member 7 [Source:HGNC Symbol;Acc:HGNC:22404] |
| ENSG00000164466 | SFXN1 | 1.3645 | 0.0060725 | sideroflexin 1 [Source:HGNC Symbol;Acc:HGNC:16085] |
| ENSG00000131797 | CLUHP3 | 1.1905 | 0.0061791 | clustered mitochondria (cluA/CLU1) homolog pseudogene 3 [Source:HGNC Symbol;Acc:HGNC:28447] |
| ENSG00000134954 | ETS1 | 1.3417 | 0.0062981 | v-ets avian erythroblastosis virus E26 oncogene homolog 1 [Source:HGNC Symbol;Acc:HGNC:3488] |
| ENSG00000211789 | TRAV12-2 | 1.9393 | 0.0063381 | T cell receptor alpha variable 12-2 [Source:HGNC Symbol;Acc:HGNC:12106] |
| ENSG00000112276 | BVES | 2.5822 | 0.0068389 | blood vessel epicardial substance [Source:HGNC Symbol;Acc:HGNC:1152] |
| ENSG00000142945 | KIF2C | 1.5776 | 0.0068389 | kinesin family member 2C [Source:HGNC Symbol;Acc:HGNC:6393] |
| ENSG00000109107 | ALDOC | 2.0002 | 0.0068442 | aldolase, fructose-bisphosphate C [Source:HGNC Symbol;Acc:HGNC:418] |
| ENSG00000141858 | SAMD1 | 0.97052 | 0.0068442 | sterile alpha motif domain containing 1 [Source:HGNC Symbol;Acc:HGNC:17958] |
| ENSG00000182866 | LCK | 1.6694 | 0.0068442 | LCK proto-oncogene, Src family tyrosine kinase [Source:HGNC Symbol;Acc:HGNC:6524] |
| ENSG00000135362 | PRR5L | 2.0079 | 0.0072361 | proline rich 5 like [Source:HGNC Symbol;Acc:HGNC:25878] |
| ENSG00000073849 | ST6GAL1 | 1.1783 | 0.007697 | ST6 beta-galactosamide alpha-2,6-sialyltranferase 1 [Source:HGNC Symbol;Acc:HGNC:10860] |
| ENSG00000089692 | LAG3 | 2.0147 | 0.0077518 | lymphocyte-activation gene 3 [Source:HGNC Symbol;Acc:HGNC:6476] |
| ENSG00000240891 | PLCXD2 | 1.6606 | 0.0077518 | phosphatidylinositol-specific phospholipase C, X domain containing 2 [Source:HGNC Symbol;Acc:HGNC:26462] |
| ENSG00000090659 | CD209 | 2.2497 | 0.0077587 | CD209 molecule [Source:HGNC Symbol;Acc:HGNC:1641] |
| ENSG00000156510 | HKDC1 | 2.612 | 0.0077587 | hexokinase domain containing 1 [Source:HGNC Symbol;Acc:HGNC:23302] |
| ENSG00000138684 | IL21 | 2.668 | 0.0078239 | interleukin 21 [Source:HGNC Symbol;Acc:HGNC:6005] |
| ENSG00000163508 | EOMES | 1.8022 | 0.0080664 | eomesodermin [Source:HGNC Symbol;Acc:HGNC:3372] |
| ENSG00000244405 | ETV5 | 1.4126 | 0.0082633 | ets variant 5 [Source:HGNC Symbol;Acc:HGNC:3494] |
| ENSG00000186193 | SAPCD2 | 1.8097 | 0.0083423 | suppressor APC domain containing 2 [Source:HGNC Symbol;Acc:HGNC:28055] |
| ENSG00000143515 | ATP8B2 | 1.3437 | 0.0084969 | ATPase, aminophospholipid transporter, class I, type 8B, member 2 [Source:HGNC Symbol;Acc:HGNC:13534] |
| ENSG00000157570 | TSPAN18 | 2.1472 | 0.0085511 | tetraspanin 18 [Source:HGNC Symbol;Acc:HGNC:20660] |
| ENSG00000197043 | ANXA6 | 1.2176 | 0.0085735 | annexin A6 [Source:HGNC Symbol;Acc:HGNC:544] |
| ENSG00000143603 | KCNN3 | 1.8832 | 0.0086124 | potassium channel, calcium activated intermediate/small conductance subfamily N alpha, member 3 [Source:HGNC Symbol;Acc:HGNC:6292] |
| ENSG00000026751 | SLAMF7 | 1.8569 | 0.008711 | SLAM family member 7 [Source:HGNC Symbol;Acc:HGNC:21394] |
| ENSG00000105122 | RASAL3 | 1.2064 | 0.0087617 | RAS protein activator like 3 [Source:HGNC Symbol;Acc:HGNC:26129] |
| ENSG00000178732 | GP5 | 2.687 | 0.009003 | glycoprotein V (platelet) [Source:HGNC Symbol;Acc:HGNC:4443] |
| ENSG00000131196 | NFATC1 | 0.9239 | 0.009345 | nuclear factor of activated T-cells, cytoplasmic, calcineurin-dependent 1 [Source:HGNC Symbol;Acc:HGNC:7775] |
| ENSG00000157833 | GAREM2 | 1.6983 | 0.009345 | GRB2 associated regulator of MAPK1 subtype 2 [Source:HGNC Symbol;Acc:HGNC:27172] |
| ENSG00000230099 | TRBV5-4 | 2.1631 | 0.0094114 | T cell receptor beta variable 5-4 [Source:HGNC Symbol;Acc:HGNC:12221] |
| ENSG00000211956 | IGHV4-34 | 2.9518 | 0.0094623 | immunoglobulin heavy variable 4-34 [Source:HGNC Symbol;Acc:HGNC:5650] |
| ENSG00000109906 | ZBTB16 | 1.6812 | 0.0095118 | zinc finger and BTB domain containing 16 [Source:HGNC Symbol;Acc:HGNC:12930] |
| ENSG00000267109 | CTD-2378E21.1 | 2.8337 | 0.0095628 | - |
| Novel00290 | -- | 2.0883 | 0.0095628 | hCG2038847, partial [Homo sapiens] |
| ENSG00000073111 | MCM2 | 1.1569 | 0.0095941 | minichromosome maintenance complex component 2 [Source:HGNC Symbol;Acc:HGNC:6944] |
| ENSG00000183918 | SH2D1A | 1.582 | 0.0096234 | SH2 domain containing 1A [Source:HGNC Symbol;Acc:HGNC:10820] |
| ENSG00000253882 | RP11-61L23.2 | 1.9553 | 0.0096234 | - |
| ENSG00000116771 | AGMAT | 1.9369 | 0.0097296 | agmatinase [Source:HGNC Symbol;Acc:HGNC:18407] |
| ENSG00000140451 | PIF1 | 1.4624 | 0.0097902 | PIF1 5'-to-3' DNA helicase [Source:HGNC Symbol;Acc:HGNC:26220] |
| ENSG00000186185 | KIF18B | 1.8015 | 0.0098708 | kinesin family member 18B [Source:HGNC Symbol;Acc:HGNC:27102] |
| ENSG00000237254 | TRBV30 | 2.0428 | 0.010287 | T cell receptor beta variable 30 (gene/pseudogene) [Source:HGNC Symbol;Acc:HGNC:12214] |
| ENSG00000197540 | GZMM | 1.9253 | 0.010416 | granzyme M [Source:HGNC Symbol;Acc:HGNC:4712] |
| ENSG00000225079 | FTH1P22 | 2.401 | 0.010428 | ferritin, heavy polypeptide 1 pseudogene 22 [Source:HGNC Symbol;Acc:HGNC:37640] |
| Novel00056 | -- | 2.3175 | 0.010652 | -- |
| ENSG00000090889 | KIF4A | 1.4294 | 0.010673 | kinesin family member 4A [Source:HGNC Symbol;Acc:HGNC:13339] |
| ENSG00000198719 | DLL1 | 1.733 | 0.010797 | delta-like 1 (Drosophila) [Source:HGNC Symbol;Acc:HGNC:2908] |
| ENSG00000085840 | ORC1 | 1.356 | 0.010906 | origin recognition complex subunit 1 [Source:HGNC Symbol;Acc:HGNC:8487] |
| ENSG00000211801 | TRAV21 | 1.9758 | 0.011003 | T cell receptor alpha variable 21 [Source:HGNC Symbol;Acc:HGNC:12118] |
| ENSG00000104970 | KIR3DX1 | 2.1796 | 0.011381 | killer cell immunoglobulin-like receptor, three domains, X1 [Source:HGNC Symbol;Acc:HGNC:25043] |
| ENSG00000129682 | FGF13 | 2.3804 | 0.01165 | fibroblast growth factor 13 [Source:HGNC Symbol;Acc:HGNC:3670] |
| ENSG00000185187 | SIGIRR | 1.2567 | 0.011714 | single immunoglobulin and toll-interleukin 1 receptor (TIR) domain [Source:HGNC Symbol;Acc:HGNC:30575] |
| ENSG00000033170 | FUT8 | 0.96168 | 0.011784 | fucosyltransferase 8 (alpha (1,6) fucosyltransferase) [Source:HGNC Symbol;Acc:HGNC:4019] |
| ENSG00000023902 | PLEKHO1 | 1.2848 | 0.012064 | pleckstrin homology domain containing O1 [Source:HGNC Symbol;Acc:HGNC:24310] |
| ENSG00000163599 | CTLA4 | 1.9794 | 0.012064 | cytotoxic T-lymphocyte-associated protein 4 [Source:HGNC Symbol;Acc:HGNC:2505] |
| ENSG00000152766 | ANKRD22 | 2.1897 | 0.012651 | ankyrin repeat domain 22 [Source:HGNC Symbol;Acc:HGNC:28321] |
| ENSG00000167633 | KIR3DL1 | 2.3392 | 0.012651 | killer cell immunoglobulin-like receptor, three domains, long cytoplasmic tail, 1 [Source:HGNC Symbol;Acc:HGNC:6338] |
| ENSG00000198771 | RCSD1 | 1.41 | 0.012742 | RCSD domain containing 1 [Source:HGNC Symbol;Acc:HGNC:28310] |
| ENSG00000159753 | RLTPR | 1.5897 | 0.012804 | RGD motif, leucine rich repeats, tropomodulin domain and proline-rich containing [Source:HGNC Symbol;Acc:HGNC:27089] |
| ENSG00000225986 | UBXN10-AS1 | 2.6204 | 0.012804 | UBXN10 antisense RNA 1 [Source:HGNC Symbol;Acc:HGNC:41141] |
| ENSG00000121152 | NCAPH | 1.5444 | 0.012852 | non-SMC condensin I complex subunit H [Source:HGNC Symbol;Acc:HGNC:1112] |
| ENSG00000175643 | RMI2 | 1.997 | 0.012852 | RecQ mediated genome instability 2 [Source:HGNC Symbol;Acc:HGNC:28349] |
| ENSG00000101187 | SLCO4A1 | 2.0645 | 0.012916 | solute carrier organic anion transporter family member 4A1 [Source:HGNC Symbol;Acc:HGNC:10953] |
| ENSG00000101412 | E2F1 | 1.2472 | 0.012916 | E2F transcription factor 1 [Source:HGNC Symbol;Acc:HGNC:3113] |
| ENSG00000148908 | RGS10 | 0.88407 | 0.012916 | regulator of G-protein signaling 10 [Source:HGNC Symbol;Acc:HGNC:9992] |
| ENSG00000131634 | TMEM204 | 1.9225 | 0.012961 | transmembrane protein 204 [Source:HGNC Symbol;Acc:HGNC:14158] |
| ENSG00000117399 | CDC20 | 1.7846 | 0.012966 | cell division cycle 20 [Source:HGNC Symbol;Acc:HGNC:1723] |
| ENSG00000211659 | IGLV3-25 | 2.7987 | 0.013301 | immunoglobulin lambda variable 3-25 [Source:HGNC Symbol;Acc:HGNC:5908] |
| ENSG00000124191 | TOX2 | 1.7825 | 0.013418 | TOX high mobility group box family member 2 [Source:HGNC Symbol;Acc:HGNC:16095] |
| ENSG00000211976 | IGHV3-73 | 2.8063 | 0.013418 | immunoglobulin heavy variable 3-73 [Source:HGNC Symbol;Acc:HGNC:5623] |
| ENSG00000167984 | NLRC3 | 1.4648 | 0.01395 | NLR family, CARD domain containing 3 [Source:HGNC Symbol;Acc:HGNC:29889] |
| ENSG00000164626 | KCNK5 | 1.8807 | 0.014055 | potassium channel, two pore domain subfamily K, member 5 [Source:HGNC Symbol;Acc:HGNC:6280] |
| ENSG00000170469 | SPATA24 | 1.2576 | 0.01413 | spermatogenesis associated 24 [Source:HGNC Symbol;Acc:HGNC:27322] |
| ENSG00000262823 | RP13-580F15.2 | 2.2677 | 0.014227 | - |
| ENSG00000211642 | IGLV10-54 | 2.8595 | 0.014406 | immunoglobulin lambda variable 10-54 [Source:HGNC Symbol;Acc:HGNC:5884] |
| ENSG00000171956 | FOXB1 | 2.4996 | 0.014626 | forkhead box B1 [Source:HGNC Symbol;Acc:HGNC:3799] |
| ENSG00000080854 | IGSF9B | 2.0668 | 0.01471 | immunoglobulin superfamily member 9B [Source:HGNC Symbol;Acc:HGNC:32326] |
| ENSG00000072694 | FCGR2B | 2.2148 | 0.014822 | Fc fragment of IgG, low affinity IIb, receptor (CD32) [Source:HGNC Symbol;Acc:HGNC:3618] |
| ENSG00000068796 | KIF2A | 0.69834 | 0.014827 | kinesin heavy chain member 2A [Source:HGNC Symbol;Acc:HGNC:6318] |
| ENSG00000066735 | KIF26A | 2.8238 | 0.01517 | kinesin family member 26A [Source:HGNC Symbol;Acc:HGNC:20226] |
| ENSG00000117394 | SLC2A1 | 1.5714 | 0.01517 | solute carrier family 2 (facilitated glucose transporter), member 1 [Source:HGNC Symbol;Acc:HGNC:11005] |
| ENSG00000135414 | GDF11 | 1.0073 | 0.01517 | growth differentiation factor 11 [Source:HGNC Symbol;Acc:HGNC:4216] |
| ENSG00000143847 | PPFIA4 | 2.2087 | 0.01517 | protein tyrosine phosphatase, receptor type, f polypeptide (PTPRF), interacting protein (liprin), alpha 4 [Source:HGNC Symbol;Acc:HGNC:9248] |
| ENSG00000172057 | ORMDL3 | 0.86311 | 0.01517 | ORMDL sphingolipid biosynthesis regulator 3 [Source:HGNC Symbol;Acc:HGNC:16038] |
| ENSG00000197635 | DPP4 | 1.5972 | 0.01517 | dipeptidyl-peptidase 4 [Source:HGNC Symbol;Acc:HGNC:3009] |
| ENSG00000235162 | C12orf75 | 1.8915 | 0.01517 | chromosome 12 open reading frame 75 [Source:HGNC Symbol;Acc:HGNC:35164] |
| ENSG00000136960 | ENPP2 | 1.9837 | 0.015554 | ectonucleotide pyrophosphatase/phosphodiesterase 2 [Source:HGNC Symbol;Acc:HGNC:3357] |
| ENSG00000115163 | CENPA | 1.4193 | 0.015591 | centromere protein A [Source:HGNC Symbol;Acc:HGNC:1851] |
| ENSG00000106565 | TMEM176B | 1.7249 | 0.015716 | transmembrane protein 176B [Source:HGNC Symbol;Acc:HGNC:29596] |
| ENSG00000104812 | GYS1 | 1.0374 | 0.016149 | glycogen synthase 1 [Source:HGNC Symbol;Acc:HGNC:4706] |
| Novel00045 | -- | 2.1646 | 0.016319 | rna-directed dna partial [Stylonychia lemnae] |
| ENSG00000176171 | BNIP3 | 1.747 | 0.016869 | BCL2/adenovirus E1B 19kDa interacting protein 3 [Source:HGNC Symbol;Acc:HGNC:1084] |
| ENSG00000237702 | TRBV3-1 | 2.3733 | 0.017065 | T cell receptor beta variable 3-1 [Source:HGNC Symbol;Acc:HGNC:12212] |
| ENSG00000100427 | MLC1 | 1.7303 | 0.017144 | megalencephalic leukoencephalopathy with subcortical cysts 1 [Source:HGNC Symbol;Acc:HGNC:17082] |
| ENSG00000161888 | SPC24 | 1.4292 | 0.017201 | SPC24, NDC80 kinetochore complex component [Source:HGNC Symbol;Acc:HGNC:26913] |
| ENSG00000189013 | KIR2DL4 | 2.3117 | 0.017446 | killer cell immunoglobulin-like receptor, two domains, long cytoplasmic tail, 4 [Source:HGNC Symbol;Acc:HGNC:6332] |
| ENSG00000130005 | GAMT | 1.6412 | 0.017938 | guanidinoacetate N-methyltransferase [Source:HGNC Symbol;Acc:HGNC:4136] |
| ENSG00000211751 | TRBC1 | 1.7703 | 0.017984 | T cell receptor beta constant 1 [Source:HGNC Symbol;Acc:HGNC:12156] |
| ENSG00000166289 | PLEKHF1 | 1.6621 | 0.018149 | pleckstrin homology and FYVE domain containing 1 [Source:HGNC Symbol;Acc:HGNC:20764] |
| ENSG00000117560 | FASLG | 1.807 | 0.01815 | Fas ligand [Source:HGNC Symbol;Acc:HGNC:11936] |
| ENSG00000125726 | CD70 | 1.4013 | 0.018235 | CD70 molecule [Source:HGNC Symbol;Acc:HGNC:11937] |
| ENSG00000247095 | MIR210HG | 2.2337 | 0.018517 | MIR210 host gene [Source:HGNC Symbol;Acc:HGNC:39524] |
| ENSG00000237943 | PRKCQ-AS1 | 1.3092 | 0.018945 | PRKCQ antisense RNA 1 [Source:HGNC Symbol;Acc:HGNC:44689] |
| ENSG00000162063 | CCNF | 1.3952 | 0.018959 | cyclin F [Source:HGNC Symbol;Acc:HGNC:1591] |
| ENSG00000167094 | TTC16 | 1.7698 | 0.019143 | tetratricopeptide repeat domain 16 [Source:HGNC Symbol;Acc:HGNC:26536] |
| ENSG00000145365 | TIFA | 1.8733 | 0.019432 | TRAF-interacting protein with forkhead-associated domain [Source:HGNC Symbol;Acc:HGNC:19075] |
| ENSG00000122188 | LAX1 | 1.5741 | 0.019636 | lymphocyte transmembrane adaptor 1 [Source:HGNC Symbol;Acc:HGNC:26005] |
| ENSG00000178498 | DTX3 | 1.5216 | 0.019636 | deltex 3, E3 ubiquitin ligase [Source:HGNC Symbol;Acc:HGNC:24457] |
| ENSG00000139182 | CLSTN3 | 1.2628 | 0.019941 | calsyntenin 3 [Source:HGNC Symbol;Acc:HGNC:18371] |
| ENSG00000143851 | PTPN7 | 1.3128 | 0.019941 | protein tyrosine phosphatase, non-receptor type 7 [Source:HGNC Symbol;Acc:HGNC:9659] |
| ENSG00000065675 | PRKCQ | 1.5143 | 0.02024 | protein kinase C, theta [Source:HGNC Symbol;Acc:HGNC:9410] |
| ENSG00000181374 | CCL13 | 1.9295 | 0.02024 | chemokine (C-C motif) ligand 13 [Source:HGNC Symbol;Acc:HGNC:10611] |
| ENSG00000276043 | UHRF1 | 1.3124 | 0.02024 | ubiquitin-like with PHD and ring finger domains 1 [Source:HGNC Symbol;Acc:HGNC:12556] |
| ENSG00000157456 | CCNB2 | 1.141 | 0.020254 | cyclin B2 [Source:HGNC Symbol;Acc:HGNC:1580] |
| ENSG00000213203 | GIMAP1 | 1.5249 | 0.020254 | GTPase, IMAP family member 1 [Source:HGNC Symbol;Acc:HGNC:23237] |
| ENSG00000198589 | LRBA | 0.75495 | 0.020313 | LPS-responsive vesicle trafficking, beach and anchor containing [Source:HGNC Symbol;Acc:HGNC:1742] |
| ENSG00000166313 | APBB1 | 1.5912 | 0.020405 | amyloid beta (A4) precursor protein-binding, family B, member 1 (Fe65) [Source:HGNC Symbol;Acc:HGNC:581] |
| ENSG00000134545 | KLRC1 | 1.9975 | 0.020494 | killer cell lectin-like receptor subfamily C, member 1 [Source:HGNC Symbol;Acc:HGNC:6374] |
| ENSG00000175063 | UBE2C | 1.4262 | 0.020865 | ubiquitin conjugating enzyme E2C [Source:HGNC Symbol;Acc:HGNC:15937] |
| ENSG00000100351 | GRAP2 | 1.4704 | 0.02104 | GRB2-related adaptor protein 2 [Source:HGNC Symbol;Acc:HGNC:4563] |
| ENSG00000090097 | PCBP4 | 1.4359 | 0.02116 | poly(rC) binding protein 4 [Source:HGNC Symbol;Acc:HGNC:8652] |
| ENSG00000211658 | IGLV3-27 | 2.7271 | 0.02116 | immunoglobulin lambda variable 3-27 [Source:HGNC Symbol;Acc:HGNC:5910] |
| ENSG00000166508 | MCM7 | 0.95943 | 0.021265 | minichromosome maintenance complex component 7 [Source:HGNC Symbol;Acc:HGNC:6950] |
| ENSG00000177542 | SLC25A22 | 0.82594 | 0.021265 | solute carrier family 25 (mitochondrial carrier: glutamate), member 22 [Source:HGNC Symbol;Acc:HGNC:19954] |
| ENSG00000100147 | CCDC134 | 0.72805 | 0.021356 | coiled-coil domain containing 134 [Source:HGNC Symbol;Acc:HGNC:26185] |
| ENSG00000104921 | FCER2 | 2.5859 | 0.021772 | Fc fragment of IgE, low affinity II, receptor for (CD23) [Source:HGNC Symbol;Acc:HGNC:3612] |
| ENSG00000108370 | RGS9 | 1.4716 | 0.02183 | regulator of G-protein signaling 9 [Source:HGNC Symbol;Acc:HGNC:10004] |
| ENSG00000148773 | MKI67 | 1.6117 | 0.021961 | marker of proliferation Ki-67 [Source:HGNC Symbol;Acc:HGNC:7107] |
| ENSG00000174327 | SLC16A13 | 1.0664 | 0.022193 | solute carrier family 16 member 13 [Source:HGNC Symbol;Acc:HGNC:31037] |
| ENSG00000115738 | ID2 | 0.9575 | 0.022304 | inhibitor of DNA binding 2, dominant negative helix-loop-helix protein [Source:HGNC Symbol;Acc:HGNC:5361] |
| ENSG00000186918 | ZNF395 | 1.6322 | 0.022304 | zinc finger protein 395 [Source:HGNC Symbol;Acc:HGNC:18737] |
| ENSG00000237649 | KIFC1 | 1.3923 | 0.022326 | kinesin family member C1 [Source:HGNC Symbol;Acc:HGNC:6389] |
| ENSG00000105255 | FSD1 | 1.5775 | 0.022558 | fibronectin type III and SPRY domain containing 1 [Source:HGNC Symbol;Acc:HGNC:13745] |
| ENSG00000233559 | AC016831.7 | 1.6518 | 0.022558 | - |
| ENSG00000048462 | TNFRSF17 | 2.4338 | 0.022672 | tumor necrosis factor receptor superfamily member 17 [Source:HGNC Symbol;Acc:HGNC:11913] |
| ENSG00000149294 | NCAM1 | 2.3018 | 0.022672 | neural cell adhesion molecule 1 [Source:HGNC Symbol;Acc:HGNC:7656] |
| ENSG00000184524 | CEND1 | 2.025 | 0.022672 | cell cycle exit and neuronal differentiation 1 [Source:HGNC Symbol;Acc:HGNC:24153] |
| ENSG00000266088 | RP5-1028K7.2 | 2.2892 | 0.022672 | - |
| ENSG00000010810 | FYN | 1.0741 | 0.022732 | FYN proto-oncogene, Src family tyrosine kinase [Source:HGNC Symbol;Acc:HGNC:4037] |
| ENSG00000111879 | FAM184A | 1.8414 | 0.0229 | family with sequence similarity 184 member A [Source:HGNC Symbol;Acc:HGNC:20991] |
| ENSG00000088325 | TPX2 | 1.3445 | 0.023003 | TPX2, microtubule-associated [Source:HGNC Symbol;Acc:HGNC:1249] |
| ENSG00000099998 | GGT5 | 2.3735 | 0.023003 | gamma-glutamyltransferase 5 [Source:HGNC Symbol;Acc:HGNC:4260] |
| ENSG00000105011 | ASF1B | 1.2745 | 0.023003 | anti-silencing function 1B histone chaperone [Source:HGNC Symbol;Acc:HGNC:20996] |
| ENSG00000105409 | ATP1A3 | 1.6237 | 0.023003 | ATPase, Na+/K+ transporting, alpha 3 polypeptide [Source:HGNC Symbol;Acc:HGNC:801] |
| ENSG00000145386 | CCNA2 | 1.3982 | 0.023119 | cyclin A2 [Source:HGNC Symbol;Acc:HGNC:1578] |
| ENSG00000166813 | KIF7 | 1.9085 | 0.023119 | kinesin family member 7 [Source:HGNC Symbol;Acc:HGNC:30497] |
| ENSG00000211637 | IGLV4-69 | 2.6202 | 0.023119 | immunoglobulin lambda variable 4-69 [Source:HGNC Symbol;Acc:HGNC:5921] |
| ENSG00000211890 | IGHA2 | 2.4957 | 0.023474 | immunoglobulin heavy constant alpha 2 (A2m marker) [Source:HGNC Symbol;Acc:HGNC:5479] |
| ENSG00000133065 | SLC41A1 | 0.69583 | 0.023826 | solute carrier family 41 (magnesium transporter), member 1 [Source:HGNC Symbol;Acc:HGNC:19429] |
| ENSG00000205809 | KLRC2 | 1.8795 | 0.024508 | killer cell lectin-like receptor subfamily C, member 2 [Source:HGNC Symbol;Acc:HGNC:6375] |
| ENSG00000185386 | MAPK11 | 0.93539 | 0.024537 | mitogen-activated protein kinase 11 [Source:HGNC Symbol;Acc:HGNC:6873] |
| ENSG00000280237 | MIR4697HG | 2.0414 | 0.024575 | MIR4697 host gene [Source:HGNC Symbol;Acc:HGNC:27448] |
| ENSG00000160654 | CD3G | 1.5067 | 0.024795 | CD3g molecule, gamma (CD3-TCR complex) [Source:HGNC Symbol;Acc:HGNC:1675] |
| ENSG00000100453 | GZMB | 2.3452 | 0.024906 | granzyme B [Source:HGNC Symbol;Acc:HGNC:4709] |
| ENSG00000211893 | IGHG2 | 2.3729 | 0.024906 | immunoglobulin heavy constant gamma 2 (G2m marker) [Source:HGNC Symbol;Acc:HGNC:5526] |
| ENSG00000160307 | S100B | 2.2682 | 0.025449 | S100 calcium binding protein B [Source:HGNC Symbol;Acc:HGNC:10500] |
| ENSG00000171603 | CLSTN1 | 0.59831 | 0.025449 | calsyntenin 1 [Source:HGNC Symbol;Acc:HGNC:17447] |
| ENSG00000224389 | C4B | 2.1494 | 0.025558 | complement component 4B (Chido blood group) [Source:HGNC Symbol;Acc:HGNC:1324] |
| ENSG00000272282 | RP11-222K16.2 | 2.2381 | 0.025681 | - |
| ENSG00000170476 | MZB1 | 2.2926 | 0.025743 | marginal zone B and B1 cell-specific protein [Source:HGNC Symbol;Acc:HGNC:30125] |
| ENSG00000124762 | CDKN1A | 1.3946 | 0.025744 | cyclin-dependent kinase inhibitor 1A (p21, Cip1) [Source:HGNC Symbol;Acc:HGNC:1784] |
| ENSG00000160957 | RECQL4 | 0.99822 | 0.025744 | RecQ helicase-like 4 [Source:HGNC Symbol;Acc:HGNC:9949] |
| ENSG00000112782 | CLIC5 | 1.4223 | 0.026227 | chloride intracellular channel 5 [Source:HGNC Symbol;Acc:HGNC:13517] |
| ENSG00000152217 | SETBP1 | 1.338 | 0.026227 | SET binding protein 1 [Source:HGNC Symbol;Acc:HGNC:15573] |
| ENSG00000140534 | TICRR | 1.5611 | 0.026895 | TOPBP1-interacting checkpoint and replication regulator [Source:HGNC Symbol;Acc:HGNC:28704] |
| ENSG00000188778 | ADRB3 | 2.5623 | 0.026895 | adrenoceptor beta 3 [Source:HGNC Symbol;Acc:HGNC:288] |
| ENSG00000172673 | THEMIS | 1.8007 | 0.02714 | thymocyte selection associated [Source:HGNC Symbol;Acc:HGNC:21569] |
| ENSG00000188820 | FAM26F | 2.3701 | 0.02714 | family with sequence similarity 26 member F [Source:HGNC Symbol;Acc:HGNC:33391] |
| ENSG00000106415 | GLCCI1 | 1.2024 | 0.027605 | glucocorticoid induced 1 [Source:HGNC Symbol;Acc:HGNC:18713] |
| ENSG00000164109 | MAD2L1 | 1.2025 | 0.027605 | MAD2 mitotic arrest deficient-like 1 (yeast) [Source:HGNC Symbol;Acc:HGNC:6763] |
| ENSG00000226979 | LTA | 1.5529 | 0.028675 | lymphotoxin alpha [Source:HGNC Symbol;Acc:HGNC:6709] |
| ENSG00000211665 | IGLV3-16 | 2.5772 | 0.028761 | immunoglobulin lambda variable 3-16 [Source:HGNC Symbol;Acc:HGNC:5901] |
| Novel00037 | -- | 1.6764 | 0.029402 | hCG2000782 [Homo sapiens] |
| ENSG00000078269 | SYNJ2 | 1.2997 | 0.029403 | synaptojanin 2 [Source:HGNC Symbol;Acc:HGNC:11504] |
| ENSG00000211895 | IGHA1 | 2.4229 | 0.029413 | immunoglobulin heavy constant alpha 1 [Source:HGNC Symbol;Acc:HGNC:5478] |
| ENSG00000264522 | OTUD7B | 0.9814 | 0.03036 | OTU deubiquitinase 7B [Source:HGNC Symbol;Acc:HGNC:16683] |
| ENSG00000211699 | TRGV3 | 1.3637 | 0.030496 | T cell receptor gamma variable 3 [Source:HGNC Symbol;Acc:HGNC:12288] |
| ENSG00000144668 | ITGA9 | 1.5937 | 0.030562 | integrin subunit alpha 9 [Source:HGNC Symbol;Acc:HGNC:6145] |
| ENSG00000150510 | FAM124A | 2.2825 | 0.030562 | family with sequence similarity 124 member A [Source:HGNC Symbol;Acc:HGNC:26413] |
| ENSG00000171843 | MLLT3 | 0.94483 | 0.030591 | myeloid/lymphoid or mixed-lineage leukemia; translocated to, 3 [Source:HGNC Symbol;Acc:HGNC:7136] |
| ENSG00000112667 | DNPH1 | 1.2151 | 0.030661 | 2'-deoxynucleoside 5'-phosphate N-hydrolase 1 [Source:HGNC Symbol;Acc:HGNC:21218] |
| ENSG00000111665 | CDCA3 | 1.2598 | 0.030693 | cell division cycle associated 3 [Source:HGNC Symbol;Acc:HGNC:14624] |
| ENSG00000100526 | CDKN3 | 1.2542 | 0.031035 | cyclin-dependent kinase inhibitor 3 [Source:HGNC Symbol;Acc:HGNC:1791] |
| ENSG00000136997 | MYC | 1.0365 | 0.031353 | v-myc avian myelocytomatosis viral oncogene homolog [Source:HGNC Symbol;Acc:HGNC:7553] |
| ENSG00000254838 | GVINP1 | 1.1812 | 0.031353 | GTPase, very large interferon inducible pseudogene 1 [Source:HGNC Symbol;Acc:HGNC:25813] |
| ENSG00000167513 | CDT1 | 1.5049 | 0.031401 | chromatin licensing and DNA replication factor 1 [Source:HGNC Symbol;Acc:HGNC:24576] |
| ENSG00000260727 | SLC7A5P1 | 1.4953 | 0.03154 | solute carrier family 7 (amino acid transporter light chain, L system), member 5 pseudogene 1 [Source:HGNC Symbol;Acc:HGNC:29458] |
| ENSG00000131153 | GINS2 | 1.3268 | 0.031694 | GINS complex subunit 2 (Psf2 homolog) [Source:HGNC Symbol;Acc:HGNC:24575] |
| ENSG00000015133 | CCDC88C | 1.0736 | 0.032066 | coiled-coil domain containing 88C [Source:HGNC Symbol;Acc:HGNC:19967] |
| ENSG00000137474 | MYO7A | 1.7271 | 0.032066 | myosin VIIA [Source:HGNC Symbol;Acc:HGNC:7606] |
| ENSG00000145779 | TNFAIP8 | 1.4589 | 0.032066 | TNF alpha induced protein 8 [Source:HGNC Symbol;Acc:HGNC:17260] |
| ENSG00000152253 | SPC25 | 1.5138 | 0.032066 | SPC25, NDC80 kinetochore complex component [Source:HGNC Symbol;Acc:HGNC:24031] |
| ENSG00000177699 | RP11-16K12.1 | 2.0396 | 0.032198 | - |
| ENSG00000142512 | SIGLEC10 | 1.3752 | 0.032552 | sialic acid binding Ig-like lectin 10 [Source:HGNC Symbol;Acc:HGNC:15620] |
| ENSG00000063176 | SPHK2 | 0.67463 | 0.033073 | sphingosine kinase 2 [Source:HGNC Symbol;Acc:HGNC:18859] |
| ENSG00000138378 | STAT4 | 1.7157 | 0.033727 | signal transducer and activator of transcription 4 [Source:HGNC Symbol;Acc:HGNC:11365] |
| ENSG00000271503 | CCL5 | 1.3307 | 0.033727 | chemokine (C-C motif) ligand 5 [Source:HGNC Symbol;Acc:HGNC:10632] |
| ENSG00000156475 | PPP2R2B | 1.8378 | 0.033781 | protein phosphatase 2 regulatory subunit B, beta [Source:HGNC Symbol;Acc:HGNC:9305] |
| ENSG00000126243 | LRFN3 | 1.6575 | 0.03387 | leucine rich repeat and fibronectin type III domain containing 3 [Source:HGNC Symbol;Acc:HGNC:28370] |
| ENSG00000134539 | KLRD1 | 1.365 | 0.03387 | killer cell lectin-like receptor subfamily D, member 1 [Source:HGNC Symbol;Acc:HGNC:6378] |
| ENSG00000091262 | ABCC6 | 1.9139 | 0.034092 | ATP binding cassette subfamily C member 6 [Source:HGNC Symbol;Acc:HGNC:57] |
| ENSG00000140525 | FANCI | 0.99666 | 0.034178 | Fanconi anemia complementation group I [Source:HGNC Symbol;Acc:HGNC:25568] |
| ENSG00000137193 | PIM1 | 1.5978 | 0.03437 | Pim-1 proto-oncogene, serine/threonine kinase [Source:HGNC Symbol;Acc:HGNC:8986] |
| ENSG00000205593 | DENND6B | 1.4353 | 0.034442 | DENN/MADD domain containing 6B [Source:HGNC Symbol;Acc:HGNC:32690] |
| ENSG00000196465 | MYL6B | 1.1841 | 0.034495 | myosin light chain 6B [Source:HGNC Symbol;Acc:HGNC:29823] |
| ENSG00000186187 | ZNRF1 | 1.3442 | 0.034683 | zinc and ring finger 1, E3 ubiquitin protein ligase [Source:HGNC Symbol;Acc:HGNC:18452] |
| ENSG00000221957 | KIR2DS4 | 2.2076 | 0.034777 | killer cell immunoglobulin-like receptor, two domains, short cytoplasmic tail, 4 [Source:HGNC Symbol;Acc:HGNC:6336] |
| ENSG00000103056 | SMPD3 | 1.2961 | 0.034985 | sphingomyelin phosphodiesterase 3 [Source:HGNC Symbol;Acc:HGNC:14240] |
| ENSG00000167912 | RP11-25K19.1 | 1.6937 | 0.034985 | - |
| ENSG00000184613 | NELL2 | 2.1747 | 0.035179 | neural EGFL like 2 [Source:HGNC Symbol;Acc:HGNC:7751] |
| ENSG00000160185 | UBASH3A | 1.5116 | 0.035628 | ubiquitin associated and SH3 domain containing A [Source:HGNC Symbol;Acc:HGNC:12462] |
| ENSG00000234261 | RP11-146I2.1 | 2.1355 | 0.035628 | - |
| ENSG00000102145 | GATA1 | 2.3491 | 0.035785 | GATA binding protein 1 (globin transcription factor 1) [Source:HGNC Symbol;Acc:HGNC:4170] |
| ENSG00000168334 | XIRP1 | 2.3167 | 0.036211 | xin actin binding repeat containing 1 [Source:HGNC Symbol;Acc:HGNC:14301] |
| ENSG00000169194 | IL13 | 2.3787 | 0.036281 | interleukin 13 [Source:HGNC Symbol;Acc:HGNC:5973] |
| ENSG00000163564 | PYHIN1 | 1.4258 | 0.036419 | pyrin and HIN domain family member 1 [Source:HGNC Symbol;Acc:HGNC:28894] |
| ENSG00000122852 | SFTPA1 | 2.5203 | 0.037318 | surfactant protein A1 [Source:HGNC Symbol;Acc:HGNC:10798] |
| ENSG00000072840 | EVC | 0.95381 | 0.037484 | Ellis van Creveld protein [Source:HGNC Symbol;Acc:HGNC:3497] |
| ENSG00000082014 | SMARCD3 | 1.29 | 0.037484 | SWI/SNF related, matrix associated, actin dependent regulator of chromatin, subfamily d, member 3 [Source:HGNC Symbol;Acc:HGNC:11108] |
| ENSG00000107742 | SPOCK2 | 1.4999 | 0.037759 | sparc/osteonectin, cwcv and kazal-like domains proteoglycan (testican) 2 [Source:HGNC Symbol;Acc:HGNC:13564] |
| ENSG00000230438 | SERPINB9P1 | 1.4459 | 0.037759 | serpin peptidase inhibitor, clade B (ovalbumin), member 9, pseudogene 1 [Source:HGNC Symbol;Acc:HGNC:28590] |
| ENSG00000171115 | GIMAP8 | 1.8745 | 0.037962 | GTPase, IMAP family member 8 [Source:HGNC Symbol;Acc:HGNC:21792] |
| ENSG00000011028 | MRC2 | 1.564 | 0.038521 | mannose receptor, C type 2 [Source:HGNC Symbol;Acc:HGNC:16875] |
| ENSG00000282122 | CH17-262H11.1 | 2.5376 | 0.038597 | - |
| ENSG00000092470 | WDR76 | 1.0389 | 0.038682 | WD repeat domain 76 [Source:HGNC Symbol;Acc:HGNC:25773] |
| ENSG00000111145 | ELK3 | 0.75074 | 0.038682 | ELK3, ETS-domain protein (SRF accessory protein 2) [Source:HGNC Symbol;Acc:HGNC:3325] |
| ENSG00000159363 | ATP13A2 | 1.2524 | 0.038682 | ATPase type 13A2 [Source:HGNC Symbol;Acc:HGNC:30213] |
| ENSG00000131142 | CCL25 | 2.3064 | 0.038875 | chemokine (C-C motif) ligand 25 [Source:HGNC Symbol;Acc:HGNC:10624] |
| ENSG00000176890 | TYMS | 1.0787 | 0.038895 | thymidylate synthetase [Source:HGNC Symbol;Acc:HGNC:12441] |
| ENSG00000182566 | CLEC4G | 2.3091 | 0.038895 | C-type lectin domain family 4 member G [Source:HGNC Symbol;Acc:HGNC:24591] |
| Novel00113 | -- | 1.8413 | 0.039918 | PREDICTED: putative uncharacterized protein C1orf136-like [Macaca mulatta] |
| ENSG00000181004 | BBS12 | 0.93653 | 0.039924 | Bardet-Biedl syndrome 12 [Source:HGNC Symbol;Acc:HGNC:26648] |
| ENSG00000211677 | IGLC2 | 2.3281 | 0.040009 | immunoglobulin lambda constant 2 (Kern-Oz- marker) [Source:HGNC Symbol;Acc:HGNC:5856] |
| ENSG00000197301 | RP11-366L20.2 | 2.3953 | 0.040015 | - |
| ENSG00000167550 | RHEBL1 | 1.4536 | 0.040181 | Ras homolog enriched in brain like 1 [Source:HGNC Symbol;Acc:HGNC:21166] |
| ENSG00000153563 | CD8A | 1.5435 | 0.041571 | CD8a molecule [Source:HGNC Symbol;Acc:HGNC:1706] |
| ENSG00000257108 | NHLRC4 | 2.2227 | 0.041581 | NHL repeat containing 4 [Source:HGNC Symbol;Acc:HGNC:26700] |
| ENSG00000105810 | CDK6 | 1.2875 | 0.042067 | cyclin-dependent kinase 6 [Source:HGNC Symbol;Acc:HGNC:1777] |
| ENSG00000189350 | FAM179A | 1.8484 | 0.042509 | family with sequence similarity 179 member A [Source:HGNC Symbol;Acc:HGNC:33715] |
| ENSG00000181104 | F2R | 1.533 | 0.043325 | coagulation factor II (thrombin) receptor [Source:HGNC Symbol;Acc:HGNC:3537] |
| ENSG00000094916 | CBX5 | 0.84026 | 0.043613 | chromobox 5 [Source:HGNC Symbol;Acc:HGNC:1555] |
| ENSG00000256553 | TRAV1-2 | 1.5353 | 0.043735 | T cell receptor alpha variable 1-2 [Source:HGNC Symbol;Acc:HGNC:12102] |
| ENSG00000100908 | EMC9 | 0.86526 | 0.043821 | ER membrane protein complex subunit 9 [Source:HGNC Symbol;Acc:HGNC:20273] |
| ENSG00000211934 | IGHV1-2 | 2.3309 | 0.043894 | immunoglobulin heavy variable 1-2 [Source:HGNC Symbol;Acc:HGNC:5550] |
| ENSG00000138080 | EMILIN1 | 1.3816 | 0.044416 | elastin microfibril interfacer 1 [Source:HGNC Symbol;Acc:HGNC:19880] |
| ENSG00000261520 | DLGAP1-AS5 | 2.3207 | 0.044416 | DLGAP1 antisense RNA 5 [Source:HGNC Symbol;Acc:HGNC:27586] |
| ENSG00000107562 | CXCL12 | 2.4131 | 0.044475 | chemokine (C-X-C motif) ligand 12 [Source:HGNC Symbol;Acc:HGNC:10672] |
| ENSG00000089685 | BIRC5 | 1.47 | 0.044576 | baculoviral IAP repeat containing 5 [Source:HGNC Symbol;Acc:HGNC:593] |
| ENSG00000079616 | KIF22 | 0.91333 | 0.045012 | kinesin family member 22 [Source:HGNC Symbol;Acc:HGNC:6391] |
| ENSG00000127191 | TRAF2 | 0.70449 | 0.045446 | TNF receptor associated factor 2 [Source:HGNC Symbol;Acc:HGNC:12032] |
| ENSG00000010030 | ETV7 | 1.7987 | 0.045554 | ets variant 7 [Source:HGNC Symbol;Acc:HGNC:18160] |
| ENSG00000168234 | TTC39C | 0.56219 | 0.045614 | tetratricopeptide repeat domain 39C [Source:HGNC Symbol;Acc:HGNC:26595] |
| ENSG00000099958 | DERL3 | 2.0041 | 0.045652 | derlin 3 [Source:HGNC Symbol;Acc:HGNC:14236] |
| ENSG00000162512 | SDC3 | 1.9221 | 0.045652 | syndecan 3 [Source:HGNC Symbol;Acc:HGNC:10660] |
| ENSG00000124212 | PTGIS | 2.2313 | 0.046712 | prostaglandin I2 (prostacyclin) synthase [Source:HGNC Symbol;Acc:HGNC:9603] |
| ENSG00000171241 | SHCBP1 | 1.2526 | 0.046712 | SHC SH2-domain binding protein 1 [Source:HGNC Symbol;Acc:HGNC:29547] |
| ENSG00000180035 | ZNF48 | 1.1161 | 0.047183 | zinc finger protein 48 [Source:HGNC Symbol;Acc:HGNC:13114] |
| ENSG00000227145 | IL21-AS1 | 1.6338 | 0.047252 | IL21 antisense RNA 1 [Source:HGNC Symbol;Acc:HGNC:40299] |
| ENSG00000171848 | RRM2 | 1.5177 | 0.047309 | ribonucleotide reductase M2 [Source:HGNC Symbol;Acc:HGNC:10452] |
| ENSG00000107281 | NPDC1 | 1.6371 | 0.047517 | neural proliferation, differentiation and control, 1 [Source:HGNC Symbol;Acc:HGNC:7899] |
| ENSG00000138180 | CEP55 | 1.2452 | 0.047733 | centrosomal protein 55kDa [Source:HGNC Symbol;Acc:HGNC:1161] |
| ENSG00000197461 | PDGFA | 1.8 | 0.047969 | platelet-derived growth factor alpha polypeptide [Source:HGNC Symbol;Acc:HGNC:8799] |
| ENSG00000057657 | PRDM1 | 1.3852 | 0.048154 | PR domain containing 1, with ZNF domain [Source:HGNC Symbol;Acc:HGNC:9346] |
| ENSG00000005448 | WDR54 | 1.7699 | 0.048596 | WD repeat domain 54 [Source:HGNC Symbol;Acc:HGNC:25770] |
| ENSG00000127564 | PKMYT1 | 1.4448 | 0.048596 | protein kinase, membrane associated tyrosine/threonine 1 [Source:HGNC Symbol;Acc:HGNC:29650] |
| ENSG00000183087 | GAS6 | 1.3531 | 0.048596 | growth arrest specific 6 [Source:HGNC Symbol;Acc:HGNC:4168] |
| ENSG00000123453 | SARDH | 1.863 | 0.048628 | sarcosine dehydrogenase [Source:HGNC Symbol;Acc:HGNC:10536] |
| ENSG00000179348 | GATA2 | 2.1183 | 0.048628 | GATA binding protein 2 [Source:HGNC Symbol;Acc:HGNC:4171] |
| ENSG00000134690 | CDCA8 | 1.1863 | 0.048697 | cell division cycle associated 8 [Source:HGNC Symbol;Acc:HGNC:14629] |
| ENSG00000152049 | KCNE4 | 2.0876 | 0.048931 | potassium channel, voltage gated subfamily E regulatory beta subunit 4 [Source:HGNC Symbol;Acc:HGNC:6244] |
| ENSG00000123838 | C4BPA | 2.2386 | 0.049134 | complement component 4 binding protein, alpha [Source:HGNC Symbol;Acc:HGNC:1325] |
| ENSG00000163359 | COL6A3 | 1.8985 | 0.049185 | collagen, type VI, alpha 3 [Source:HGNC Symbol;Acc:HGNC:2213] |
| ENSG00000174326 | SLC16A11 | 2.1517 | 0.049185 | solute carrier family 16 member 11 [Source:HGNC Symbol;Acc:HGNC:23093] |
| ENSG00000196839 | ADA | 0.84911 | 0.04924 | adenosine deaminase [Source:HGNC Symbol;Acc:HGNC:186] |
| ENSG00000111206 | FOXM1 | 1.377 | 0.04958 | forkhead box M1 [Source:HGNC Symbol;Acc:HGNC:3818] |
| ENSG00000034053 | APBA2 | 1.8615 | 0.049905 | amyloid beta (A4) precursor protein-binding, family A, member 2 [Source:HGNC Symbol;Acc:HGNC:579] |
| ENSG00000135127 | CCDC64 | 1.3178 | 0.049905 | coiled-coil domain containing 64 [Source:HGNC Symbol;Acc:HGNC:28095] |
