# Supplementary material for: Transcriptome Analysis of Bronchoalveolar Lavage Fluid From Children With Mycoplasma pneumoniae Pneumonia Reveals Natural Killer and T Cell-Proliferation Responses
Source: Front Immunol. 2018 Jun 18;9:1403. doi: 10.3389/fimmu.2018.01403 (PMC6015898; doi:10.3389/fimmu.2018.01403)
Supplement: Supplementary file 8 [file table_6.doc]

**Additional File 6: Table S6. Top 30 gene ontology distributions of the differentially expressed genes between MPP group and control group**

| GO accession | Description | Up | up Gene name | Down | down Gene name | Over represented p-Value |
| --- | --- | --- | --- | --- | --- | --- |
| GO:0042127 | regulation of cell proliferation | 8 | MYC|CXCL12|RASAL3|CARD11|EGLN3|RLTPR|ETV5|ST6GAL1 | 4 | RBP4|CCPG1|CCDC88A|LAMB1 | 9.02E-05 |
| GO:0008283 | cell proliferation | 9 | CARD11|CXCL12|RASAL3|MYC|ETV5|TNFRSF17|ST6GAL1|RLTPR|EGLN3 | 4 | RBP4|LAMB1|CCDC88A|CCPG1 | 0.000167 |
| GO:0008284 | positive regulation of cell proliferation | 7 | RASAL3|CXCL12|CARD11|MYC|ST6GAL1|ETV5|RLTPR | 2 | LAMB1|CCPG1 | 0.000294 |
| GO:0032946 | positive regulation of mononuclear cell proliferation | 4 | RLTPR|CARD11|ST6GAL1|RASAL3 | 0 | - | 0.00038 |
| GO:0070665 | positive regulation of leukocyte proliferation | 4 | ST6GAL1|RASAL3|CARD11|RLTPR | 0 | - | 0.00038 |
| GO:0023051 | regulation of signaling | 11 | BVES|EVC|CARD11|RASAL3|CLSTN1|KIF7| OXCT1|IL18BP|SH2D1A|CXCL12|MYC | 5 | OCRL|CHRNA3|KCNC4|RBP4|ANKRD6 | 0.000393 |
| GO:0007274 | neuromuscular synaptic transmission | 1 | ETV5 | 2 | CHRNA3|DTNA | 0.000442 |
| GO:0032944 | regulation of mononuclear cell proliferation | 4 | ST6GAL1|RASAL3|CARD11|RLTPR | 0 | - | 0.00059 |
| GO:0070663 | regulation of leukocyte proliferation | 4 | RASAL3|ST6GAL1|CARD11|RLTPR | 0 | - | 0.00059 |
| GO:0048522 | positive regulation of cellular process | 14 | CXCL12|SH2D1A|MYC|ST6GAL1|RLTPR|EVC| CARD11|RASAL3|CLSTN1|OXCT1|KIF7|ETV5|BVES|EGLN3 | 7 | RBP4|ANKRD6|CALCOCO1|CCDC88A|CCPG1|LAMB1|CHRNA3 | 0.000617 |
| GO:0007275 | multicellular organismal development | 11 | TNFRSF17|ETV5|BVES|EVC|CARD11|CLSTN1 |RASAL3|OXCT1|RLTPR|CXCL12|MYC | 10 | OCRL|LAMB1|CHRNA3|CPE|RBM11|MME|CCDC88A|RBP4|ROBO3|ANKRD6 | 0.000639 |
| GO:0098602 | single organism cell adhesion | 6 | ST6GAL1|CXCL12|RASAL3|CARD11|BVES| RLTPR | 1 | LAMB1 | 0.00071 |
| GO:0034446 | substrate adhesion-dependent cell spreading | 2 | ST6GAL1|BVES | 1 | LAMB1 | 0.00094 |
| GO:0048518 | positive regulation of biological process | 15 | BVES|EGLN3|FCER2|ETV5|OXCT1|KIF7|CARD11|EVC|RASAL3|CLSTN1|RLTPR|ST6GAL1|MYC|CXCL12|SH2D1A | 8 | OCRL|LAMB1|CHRNA3|CALCOCO1|CCDC88A|CCPG1|ANKRD6|RBP4 | 0.00096 |
| GO:0023052 | signaling | 16 | SH2D1A|CXCL12|MYC|IL18BP|RLTPR|RASAL3|CLSTN1|EVC|CARD11|ATP1A3|KIF7|OXCT1|ETV5|FCER2|TNFRSF17|BVES | 10 | RBP4|KCNC4|MAST2|ANKRD6|CCDC88A|CALCOCO1|OCRL|CHRNA3|DTNA|CPE | 0.000982 |
| GO:0044700 | single organism signaling | 16 | RASAL3|CLSTN1|CARD11|EVC|ATP1A3|KIF7|OXCT1|ETV5|FCER2|TNFRSF17|BVES|SH2D1A|CXCL12|MYC|IL18BP|RLTPR | 10 | CHRNA3|OCRL|DTNA|CPE|RBP4|KCNC4|MAST2|ANKRD6|CCDC88A|CALCOCO1 | 0.000982 |
| GO:0044767 | single-organism developmental process | 12 | BVES|TNFRSF17|ETV5|OXCT1|CARD11|EVC|CLSTN1|RASAL3|RLTPR|ST6GAL1|MYC|CXCL12 | 11 | CPE|LAMB1|OCRL|CHRNA3|RBM11|MME|CCDC88A|ANKRD6|MAST2|ROBO3|RBP4 | 0.00117 |
| GO:0007626 | locomotory behavior | 3 | ETV5|ATP1A3|CXCL12 | 1 | CHRNA3 | 0.001203 |
| GO:0051716 | cellular response to stimulus | 15 | RLTPR|IL18BP|SH2D1A|CXCL12|MYC|ETV5|TNFRSF17|FCER2|EGLN3|CARD11|EVC|ATP1A3|RASAL3|KIF7|OXCT1 | 13 | CCDC88A|MME|CALCOCO1|RBP4|MAST2|ANKRD6|DENND4C|CHRNA3|OCRL|CPE|DTNA|AOC2|RBM11 | 0.001334 |
| GO:0003206 | cardiac chamber morphogenesis | 0 | - | 2 | CPE|RBP4 | 0.001495 |
| GO:0051239 | regulation of multicellular organismal process | 8 | ETV5|RLTPR|BVES|ATP1A3|CARD11|CLSTN1|CXCL12|MYC | 5 | CHRNA3|CCDC88A|RBP4|MAST2|ANKRD6 | 0.001599 |
| GO:0032502 | developmental process | 12 | RLTPR|ST6GAL1|MYC|CXCL12|BVES|ETV5|TNFRSF17|OXCT1|CLSTN1|RASAL3|CARD11|EVC | 11 | CCDC88A|MME|MAST2|ANKRD6|ROBO3|RBP4|CPE|CHRNA3|LAMB1|OCRL|RBM11 | 0.001601 |
| GO:0050793 | regulation of developmental process | 8 | ST6GAL1|ETV5|BVES|RLTPR|CXCL12|CLSTN1|CARD11|MYC | 4 | CHRNA3|CCDC88A|RBP4| ANKRD6 | 0.001737 |
| GO:0032943 | mononuclear cell proliferation | 4 | RLTPR|CARD11|ST6GAL1|RASAL3 | 0 | - | 0.001787 |
| GO:0070661 | leukocyte proliferation | 4 | RLTPR|CARD11|RASAL3|ST6GAL1 | 0 | - | 0.001787 |
| GO:2000026 | regulation of multicellular organismal development | 6 | MYC|CXCL12|CLSTN1|CARD11|RLTPR|ETV5 | 4 | ANKRD6|RBP4|CCDC88A| CHRNA3 | 0.001793 |
| GO:0042995 | cell projection | 5 | ATP1A3|EVC|CLSTN1|KIF7|BVES | 6 | KCNC4|OCRL|CHRNA3|MME|CCDC88A|DTNA | 0.000937 |
| GO:0044459 | plasma membrane part | 9 | RLTPR|BVES|FCER2|EVC|ATP1A3|CARD11|CXCL12|CLSTN1|RASAL3 | 5 | MME|CPE|DTNA|CHRNA3| KCNC4 | 0.00142 |
| GO:0045202 | synapse | 2 | CLSTN1|ATP1A3 | 5 | MME|DTNA|CPE|CHRNA3| KCNC4 | 0.001626 |
| GO:0016638 | oxidoreductase activity, acting on the CH-NH2 group of donors | 0 | - | 2 | AOC2|Novel00074 | 0.001692 |
