# Supplementary material for: Transcriptome Analysis of Bronchoalveolar Lavage Fluid From Children With Mycoplasma pneumoniae Pneumonia Reveals Natural Killer and T Cell-Proliferation Responses
Source: Front Immunol. 2018 Jun 18;9:1403. doi: 10.3389/fimmu.2018.01403 (PMC6015898; doi:10.3389/fimmu.2018.01403)
Supplement: Supplementary file 9 [file table_7.doc]

| **Additional File 7: Table S7.** Directed Acyclic Graph (DAG) of deferentially enriched GO terms of up-regulation genes. | | | | | |
| --- | --- | --- | --- | --- | --- |
| GO accession | Description | padj | DEG item | Bg item | Gene name |
| GO:0032946 | positive regulation of mononuclear cell proliferation | 0.007402 | 4 | 10 | ST6GAL1|CARD11|RLTPR|RASAL3 |
| GO:0070665 | positive regulation of leukocyte proliferation | 0.007402 | 4 | 10 | RASAL3|CARD11|ST6GAL1|RLTPR |
| GO:0032944 | regulation of mononuclear cell proliferation | 0.007402 | 4 | 11 | RLTPR|ST6GAL1|CARD11|RASAL3 |
| GO:0070663 | regulation of leukocyte proliferation | 0.007402 | 4 | 11 | CARD11|ST6GAL1|RLTPR|RASAL3 |
| GO:0048584 | positive regulation of response to stimulus | 0.007402 | 10 | 132 | SH2D1A|RASAL3|RLTPR|EVC|MYC|CARD11|KIF7|FCER2|OXCT1|CXCL12 |
| GO:0002376 | immune system process | 0.007402 | 11 | 180 | CXCL12|CARD11|ST6GAL1|FCER2|BVES|IL18BP|MYC|RLTPR|TNFRSF17|SH2D1A|RASAL3 |
| GO:0023051 | regulation of signaling | 0.007402 | 11 | 187 | IL18BP|EVC|MYC|SH2D1A|RASAL3|CXCL12|OXCT1|KIF7|CARD11|CLSTN1|BVES |
| GO:0048583 | regulation of response to stimulus | 0.007402 | 12 | 232 | RLTPR|RASAL3|SH2D1A|IL18BP|MYC|EVC|FCER2|KIF7|CARD11|ST6GAL1|OXCT1| CXCL12 |
| GO:0048522 | positive regulation of cellular process | 0.007402 | 14 | 304 | RASAL3|SH2D1A|RLTPR|EVC|MYC|ETV5|ST6GAL1|CLSTN1|KIF7|CARD11|BVES|CXCL12|EGLN3|OXCT1 |
| GO:0048518 | positive regulation of biological process | 0.007402 | 15 | 364 | SH2D1A|RASAL3|RLTPR|EVC|MYC|CARD11|KIF7|FCER2|OXCT1|CXCL12|ST6GAL1| CARD11|CXCL12|EGLN3|OXCT1 |
| GO:0023052 | signaling | 0.007402 | 16 | 447 | EVC|IL18BP|TNFRSF17|SH2D1A|KIF7|BVES|MYC|ETV5|ATP1A3|RASAL3|RLTPR|CXCL12|OXCT1|CLSTN1|CARD11|FCER2 |
| GO:0044700 | single organism signaling | 0.007402 | 16 | 447 | TNFRSF17|SH2D1A|IL18BP|EVC|KIF7|BVES|RLTPR|ATP1A3|RASAL3|ETV5|MYC|CLSTN1|CARD11|FCER2|OXCT1|CXCL12 |
| GO:0008284 | positive regulation of cell proliferation | 0.009517 | 7 | 64 | MYC|CXCL12|ETV5|RASAL3|CARD11|ST6GAL1|RLTPR |
| GO:0008283 | cell proliferation | 0.010526 | 9 | 124 | MYC|ETV5|TNFRSF17|RASAL3|RLTPR|EGLN3|CXCL12|CARD11|ST6GAL1 |
| GO:0042127 | regulation of cell proliferation | 0.014824 | 8 | 99 | CARD11|ST6GAL1|CXCL12|EGLN3|RASAL3|RLTPR|MYC|ETV5 |
| GO:0032943 | mononuclear cell proliferation | 0.017016 | 4 | 15 | RASAL3|CARD11|ST6GAL1|RLTPR |
| GO:0070661 | leukocyte proliferation | 0.017016 | 4 | 15 | RASAL3|CARD11|ST6GAL1|RLTPR |
| DEG item: Differentially expressed genes numbers correlation with the GO term | | | | | |
| Bg item: Background genes numbers correlation with the GO term | | | | | |
| padj: adjusted p value | | | | | |
