# Supplementary material for: Transcriptome Analysis of Bronchoalveolar Lavage Fluid From Children With Mycoplasma pneumoniae Pneumonia Reveals Natural Killer and T Cell-Proliferation Responses
Source: Front Immunol. 2018 Jun 18;9:1403. doi: 10.3389/fimmu.2018.01403 (PMC6015898; doi:10.3389/fimmu.2018.01403)
Supplement: Supplementary file 10 [file table_8.doc]

| **Additional File 8: Table S8.** KEGG pathway enrichment. | | | | |
| --- | --- | --- | --- | --- |
| Term | ID | padj | Input | KEGG_ID/KO |
| T cell receptor signaling pathway | hsa04660 | 0.000688792 | CD3E|GADS(GRAP2) |NFAT(NFATC1) |CD3Z(CD247) |FYN|P38(MAPK11) |IFNG|ITK|ZAP70|CD3D|NFAT(NFATC2) |CD8(CD8A) |LCK|CD3G | hsa:916|hsa:9402|hsa:4772|hsa:919|hsa:2534|hsa:5600|hsa:3458|hsa:3702|hsa:7535|hsa:915|hsa:4773|hsa:925|hsa:3932|hsa:917 |
| Natural killer cell mediated cytotoxicity | hsa04650 | 0.002757134 | Perforin(PRF1) |SAP(SH2D1A) | NFATC1|CD94(KLRD1) |FYN| IFNG|NKG2C(KLRC2) |Granzyme (GZMB)|NFATC2|ZAP70|FASL|LCK|KIR2DS(KIR2DS4) |CD3Z(CD247) | hsa:5551|hsa:4068|hsa:4772|hsa:3811|hsa:3824|hsa:2534|hsa:3804|hsa:3458|hsa:3821|hsa:3002|hsa:4773|hsa:7535|hsa:356|hsa:3932|hsa:3809|hsa:919|hsa:3822|hsa:3805 |
| Hematopoietic cell lineage | hsa04640 | 0.031066816 | CD3E|CD25(IL2RA) |CD3G|CD8(CD8A) | CD3D|CD2|CD7 | hsa:916|hsa:3559|hsa:917|hsa:925|hsa:915|hsa:914|hsa:924 |
| padj: adjusted p value | | | | |
