# Supplementary material for: Transcriptome Analysis of Bronchoalveolar Lavage Fluid From Children With Mycoplasma pneumoniae Pneumonia Reveals Natural Killer and T Cell-Proliferation Responses
Source: Front Immunol. 2018 Jun 18;9:1403. doi: 10.3389/fimmu.2018.01403 (PMC6015898; doi:10.3389/fimmu.2018.01403)
Supplement: Supplementary file 11 [file table_9.doc]

| **Additional File 10: Table S9. Clinical characteristics of the patients involved in qRT-PCR.** | |  |
| --- | --- | --- |
|  | Control | MPP |
|  | (n = 10) | (n = 18) |
| Gender (female/male) | 4/6 | 8/10 |
| Median age (range), years | 1.8 (0.92 -2.75) | 3 (0.92 - 9) |
| Fever duration, days | - | 10.83 ± 5.12 |
| Glucocorticoid (yes/no) | 0/10 | 16/14 |
| Pleural effusion (yes/no) | 0/10 | 3/15 |
| Extrapulmonary manifestations (yes/no) | 0/10 | 5/13 |
| Median CRP (range), mg/L | - | 9.52 (0.7 - 36) |
| Peripheral blood cell count |  |  |
| Blood leukocyte count (×10^9 cells/L) | - | 9.02 ± 3.78 |
| Blood monocyte count (%) | - | 7 (3 - 11) |
| Blood neutrophil count (%) | - | 53 (29 - 86) |
| Blood lymphocyte count (%) | - | 35 (11 - 63) |
| Blood eosinophil count (%) | - | 2 (0 - 5) |
| Indication of bronchoscopy |  |  |
| Diagnosis and remove of bronchus foreign body | 8 | 0 |
| Re-examination of bronchus foreign body | 2 | 0 |
| Diagnosis and treatment of lobar pneumonia | 0 | 10 |
| Diagnosis and treatment of segmental pneumonia | 0 | 8 |
| Timing of BALF collection |  |  |
| Before treatment | 10 | 18 |
| After the treatment | 0 | 0 |
| Quantitative data with a normal distribution are presented as mean ± SD. Quantitative data with a non-normal distribution are presented as median (IQR). | | |
