# Supplementary material for: Transcriptome Analysis of Bronchoalveolar Lavage Fluid From Children With Mycoplasma pneumoniae Pneumonia Reveals Natural Killer and T Cell-Proliferation Responses
Source: Front Immunol. 2018 Jun 18;9:1403. doi: 10.3389/fimmu.2018.01403 (PMC6015898; doi:10.3389/fimmu.2018.01403)
Supplement: Supplementary file 12 [file table_10.doc]

**Additional File 11: Table S10. Primers used in qRT-PCR**

|  | Gene name |  | Sequence (5' -> 3') | Amplicon Size | Length | Tm |
| --- | --- | --- | --- | --- | --- | --- |
| 1 | CD3D (CD3 delta) | Forward Primer | ACTGGCTACCCTTCTCTCG | 110 | 19 | 60.1 |
|  |  | Reverse Primer | CCGTTCCCTCTACCCATGTGA |  | 21 | 63.0 |
| 2 | CD3E (CD3 epsilon) | Forward Primer | TGCTGCTGGTTTACTACTGGA | 141 | 21 | 60.8 |
|  |  | Reverse Primer | GGATGGGCTCATAGTCTGGG |  | 20 | 61.2 |
| 3 | CD3G (CD3 gamma) | Forward Primer | GGAATCTGGGAAGTAATGCCAA | 112 | 22 | 60.0 |
|  |  | Reverse Primer | TCAATGCAGTTCTGACACATTCT |  | 23 | 60.2 |
| 4 | CD3Z(CD247) | Forward Primer | GCCAGAACCAGCTCTATAACG | 75 | 21 | 60.2 |
|  |  | Reverse Primer | GGCCACGTCTCTTGTCCAA |  | 19 | 61.9 |
| 5 | CD8A | Forward Primer | ATGGCCTTACCAGTGACCG | 104 | 19 | 61.4 |
|  |  | Reverse Primer | AGGTTCCAGGTCCGATCCAG |  | 20 | 62.9 |
| 6 | LCK | Forward Primer | TGCCATTATCCCATAGTCCCA | 95 | 21 | 60.1 |
|  |  | Reverse Primer | GAGCCTTCGTAGGTAACCAGT |  | 21 | 60.9 |
| 7 | FYN | Forward Primer | TGGAGGTGTGAACTCTTCGTC | 102 | 21 | 61.4 |
|  |  | Reverse Primer | TCTGTCCGTGCTTCATAGTCA |  | 21 | 60.6 |
| 8 | ZAP70 | Forward Primer | CGAGCGTGTATGAGAGCCC | 82 | 19 | 62.2 |
|  |  | Reverse Primer | ATGAGGAGGTTATCGCGCTTC |  | 21 | 62.0 |
| 9 | IFNG | Forward Primer | TCGGTAACTGACTTGAATGTCCA | 93 | 23 | 61.2 |
|  |  | Reverse Primer | TCGCTTCCCTGTTTTAGCTGC |  | 21 | 62.9 |
| 10 | FASL | Forward Primer | ATTTAACAGGCAAGTCCAACTCA | 99 | 23 | 60.1 |
|  |  | Reverse Primer | GGCCACCCTTCTTATACTTCACT |  | 23 | 61.4 |
| 11 | Perforin (PRF1) | Forward Primer | GTGGGACAATAACAACCCCAT | 212 | 21 | 60.0 |
|  |  | Reverse Primer | TGGCATGATAGCGGAATTTTAGG |  | 23 | 60.7 |
| 12 | Granzyme (GZMB) | Forward Primer | CCCTGGGAAAACACTCACACA | 110 | 21 | 62.2 |
|  |  | Reverse Primer | GCACAACTCAATGGTACTGTCG |  | 22 | 61.4 |
| 13 | CD94 (KLRD1) | Forward Primer | CAGGACCCAACATAGAACTCCA | 92 | 22 | 61.1 |
|  |  | Reverse Primer | GGAAATGAAGTAACAGTTGCACC |  | 23 | 60.0 |
| 14 | CD2 | Forward Primer | ACCTGTGAGGTAATGAATGGAAC | 101 | 23 | 60.0 |
|  |  | Reverse Primer | GTGGTCCACTTGTGTGTGATG |  | 21 | 61.1 |
| 15 | CD7 | Forward Primer | GCCTCCGTCAACATCACCTG | 104 | 20 | 62.8 |
|  |  | Reverse Primer | ACCCCGTCCTCGTAGTAAATG |  | 21 | 61.0 |
| 16 | CD25 (IL2RA) | Forward Primer | CGCAGAATAAAAAGCGGGTCA | 116 | 21 | 61.2 |
|  |  | Reverse Primer | ACTTGTTTCGTTGTGTTCCGA |  | 21 | 60.4 |
| 17 | P38 (MAPK11) | Forward Primer | CTGAACAACATCGTCAAGTGCC | 211 | 22 | 62.0 |
|  |  | Reverse Primer | CATAGCCGGTCATCTCCTCG |  | 20 | 61.6 |
| 18 | NFATC1 | Forward Primer | CACCGCATCACAGGGAAGAC | 119 | 20 | 62.8 |
|  |  | Reverse Primer | GCACAGTCAATGACGGCTC |  | 19 | 61.1 |
| 19 | NFATC2 | Forward Primer | GAGCCGAATGCACATAAGGTC | 107 | 21 | 61.1 |
|  |  | Reverse Primer | CCAGAGAGACTAGCAAGGGG |  | 20 | 60.7 |
| 20 | GADS (GRAP2) | Forward Primer | CTCCATCTCTGTCAGGCATGA | 127 | 21 | 61.0 |
|  |  | Reverse Primer | TGTCCTGTAGTAGTCTACCAGC |  | 22 | 60.0 |
| 21 | ITK | Forward Primer | GAAGATCGTCATGGGAAGAAGC | 114 | 22 | 60.7 |
|  |  | Reverse Primer | CGGGTATTTATAGTGGCATGGG |  | 22 | 60.3 |
| 22 | SAP (SH2D1A) | Forward Primer | AGGCGTGTACTGCCTATGTG | 183 | 20 | 61.7 |
|  |  | Reverse Primer | TGCAGAGGTATTACAATGCCTTG |  | 23 | 60.6 |
| 23 | NKG2C (KLRC2) | Forward Primer | GCCAGCATTTTACCTTCCTCA | 131 | 21 | 60.3 |
|  |  | Reverse Primer | ACTGCACAGTTAAGTTCAGCAT |  | 22 | 60.2 |
| 24 | KIR2DS (KIR2DS4) | Forward primer | GTGACCCTCTGGACATGGTG | 128 | 20 | 60.0 |
|  |  | Reverse primer | CATGTCATAGGAGCTCCGGG |  | 20 | 59.7 |
| 25 | GAPDH | Forward primer | ACAACTTTGGTATCGTGGAAGG | 101 | 22 | 60.2 |
|  |  | Reverse primer | GCCATCACGCCACAGTTTC |  | 19 | 61.7 |
