# Supplementary material for: Transcriptome Analysis of Bronchoalveolar Lavage Fluid From Children With Mycoplasma pneumoniae Pneumonia Reveals Natural Killer and T Cell-Proliferation Responses
Source: Front Immunol. 2018 Jun 18;9:1403. doi: 10.3389/fimmu.2018.01403 (PMC6015898; doi:10.3389/fimmu.2018.01403)
Supplement: Supplementary file 13 [file table_11.doc]

**Additional File 13: Table S11. Summary of the differentially expressed genes with alternative splicing.**

| AS  events | ID | GeneID | Gene name | chr | strand | exonStart_0base | exonEnd | upstreamES | upstreamEE | downstreamES | downstreamEE | FDR | IncLevel Difference |
| --- | --- | --- | --- | --- | --- | --- | --- | --- | --- | --- | --- | --- | --- |
| Skipped exon | 49886 | ENSG00000125246 |  | 13 | + | 99862990 | 99863092 | 99858860 | 99859049 | 99864817 | 99864911 | 0.000736 | -0.047 |
|  | 67451 | ENSG00000172113 |  | 3 | - | 48298426 | 48298523 | 48295144 | 48295235 | 48301278 | 48301367 | 0.000736 | -0.02 |
|  | 9116 | ENSG00000118162 |  | 19 | - | 47483294 | 47483379 | 47483160 | 47483215 | 47483934 | 47484085 | 0.000736 | 0.145 |
|  | 42406 | ENSG00000165457 |  | 11 | + | 72217336 | 72217423 | 72216793 | 72216925 | 72218560 | 72218734 | 0.002329 | -0.311 |
|  | 18744 | ENSG00000102984 |  | 16 | - | 71864902 | 71865048 | 71864137 | 71864242 | 71867911 | 71868037 | 0.004147 | 0.02 |
|  | 52385 | ENSG00000162885 |  | 1 | - | 235465635 | 235465714 | 235455558 | 235455684 | 235470849 | 235470930 | 0.004321 | -0.036 |
|  | 64272 | ENSG00000141337 |  | 17 | + | 68395072 | 68395193 | 68385063 | 68385172 | 68401359 | 68401450 | 0.005833 | -0.025 |
|  | 37383 | ENSG00000022267 | FHL1 | X | + | 136208454 | 136208641 | 136207791 | 136207961 | 136209870 | 136209954 | 0.007038 | 0.006 |
|  | 35117 | ENSG00000168421 | RHOH | 4 | + | 40200511 | 40200565 | 40197033 | 40197300 | 40242713 | 40242834 | 0.009648 | -0.291 |
|  | 26716 | ENSG00000126214 |  | 14 | + | 103687080 | 103687211 | 103679383 | 103679518 | 103693552 | 103693953 | 0.010701 | -0.017 |
|  | 29590 | ENSG00000139629 |  | 12 | - | 51390849 | 51390937 | 51383460 | 51383582 | 51391081 | 51391195 | 0.011475 | -0.043 |
|  | 49096 | ENSG00000138050 |  | 2 | - | 39755294 | 39755409 | 39744369 | 39744478 | 39755888 | 39755960 | 0.011945 | -0.031 |
|  | 48812 | ENSG00000109944 |  | 11 | + | 122924896 | 122925010 | 122903924 | 122904444 | 122934419 | 122935075 | 0.025812 | 0.132 |
|  | 46129 | ENSG00000029725 |  | 17 | + | 5381388 | 5381505 | 5378176 | 5378232 | 5383121 | 5385812 | 0.028875 | -0.061 |
|  | 41703 | ENSG00000140105 |  | 14 | - | 100374135 | 100374265 | 100369086 | 100369258 | 100375510 | 100375607 | 0.032801 | 0.454 |
|  | ID | GeneID |  | chr | strand | riExonStart_0base | riExonEnd | upstreamES | upstreamEE | downstreamES | downstreamEE | FDR | IncLevel Difference |
| Retained intron | 1647 | ENSG00000170889 |  | 19 | + | 54206552 | 54207647 | 54206552 | 54206724 | 54207397 | 54207647 | 1.11E-06 | -0.158 |
|  | 541 | ENSG00000131042 |  | 19 | - | 54276806 | 54277600 | 54276806 | 54276929 | 54277549 | 54277600 | 2.95E-06 | -0.323 |
|  | 5914 | ENSG00000101460 |  | 20 | + | 34559735 | 34560345 | 34559735 | 34559905 | 34560171 | 34560345 | 0.023563 | -0.201 |
|  | 3633 | ENSG00000115523 | GNLY | 2 | + | 85694752 | 85695423 | 85694752 | 85694996 | 85695319 | 85695423 | 0.034133 | -0.308 |
|  | 5062 | ENSG00000069399 |  | 19 | + | 44758245 | 44758841 | 44758245 | 44758473 | 44758723 | 44758841 | 0.036847 | -0.157 |
|  | ID | GeneID |  | chr | strand | longExonStart 0base | longExonEnd | shortES | shortEE | flankingES | flankingEE | FDR | IncLevel Difference |
| A5SS | 468 | ENSG00000163138 |  | 4 | + | 20700458 | 20700747 | 20700458 | 20700617 | 20704465 | 20704533 | 0.00244 | 0.259 |
|  | ID | GeneID |  | chr | strand | longExonStart 0base | longExonEnd | shortES | shortEE | flankingES | flankingEE | FDR | IncLevel Difference |
| A3SS | 3305 | ENSG00000018280 | SLC11A1 | 2 | + | 218392114 | 218393130 | 218392980 | 218393130 | 218391375 | 218391495 | 1.98E-07 | -0.135 |
